# Mesostructure, Toolpaths and the Finite-Element Prediction Gap in the Flexural Response of Planar and Non-Planar Fused Filament Fabrication

**DOI:** 10.3390/polym18202604

**Published:** 2026-10-21

**Authors:** Ivan Grgić, Jubayer Ahmed Sajid, Jure Marijić, Mirko Karakašić

**Affiliations:** 1 Mechanical Engineering Faculty in Slavonski Brod, University of Slavonski Brod, 35000 Slavonski Brod, Croatia; jmarijic@unisb.hr (J.M.); mkarakasic@unisb.hr (M.K.); 2 Faculty of Aerospace Engineering, Aviation and Aerospace University, Bangladesh, Lalmonirhat 5500, Bangladesh; sajid-22014029@aaub.edu.bd

**Keywords:** fused filament fabrication, polylactic acid, flexural strength, crystallinity, annealing, hygrothermal ageing, non-planar toolpaths, mesostructure, finite element analysis, interlayer bonding

## Abstract

The flexural response of a fused filament fabrication (FFF) part depends on the polymer, on the mesostructure the toolpath builds from it, and on how that mesostructure is oriented to the bending load. This review examines these factors for polylactic acid (PLA), polyethylene terephthalate glycol (PETG) and acrylonitrile butadiene styrene (ABS) printed with planar and non-planar toolpaths, and how well finite-element models predict the result. Because the interlayer bond forms while the polymer cools, crystallinity, annealing, moisture, ageing and test temperature shift flexural strength and stiffness, in directions that differ between polymers and treatment windows. The toolpath is a primary mechanical variable, with effects that depend on the test conditions. Of 84 finite-element studies, 26 are compared quantitatively with a flexural experiment, 23 independently. Only 1 of those 26 treats curved-layer or non-planar deposition, and the shortfall lies in the small number of non-planar studies that predict a mechanical response, not in a lower standard of validation. None of the validated models makes the polymer constants depend on temperature, moisture or loading rate, or gives the bond between layers of one polymer its own interface law, leaving thermal, hygroscopic and time-dependent behaviour, with non-planar bending, as the clearest gaps.

## 1. Introduction

Material-extrusion additive manufacturing, known as fused deposition modelling (FDM) or fused filament fabrication (FFF), has passed from a rapid-prototyping technique into a route for functional, mechanically loaded parts. It now produces orthopaedic and orthotic devices [1,2,3], structural parts and forming tools [4,5], lightweight lattices and sandwich cores [6,7,8], parts with embedded sensing or actuation [9,10], and tissue-mimicking phantoms for needle insertion [11]. Many of these, such as orthoses, prosthetic shanks, wing structures and sandwich cores, carry their service loads in bending and not in pure tension. The flexural response is therefore the property that most often decides whether a printed part is fit for purpose, and it is also among the most demanding to control, since it is governed by the outer fibres where the stress is highest and where the printed mesostructure is most exposed. A consequence reported across the recent literature is that parts printed from the same batch of filament can differ widely in bending strength and stiffness according only to how they were built [12,13].

The reason lies in the way the process builds material. A part is printed as extruded material laid side by side within a layer and stacked layer upon layer, so the finished solid is not a homogeneous polymer but a bonded assembly with voids at the junctions [14,15]. The extrusion takes a roughly rectangular or elliptical cross-section rather than a square one, so a void area remains where each layer meets its neighbours, and even a nominally solid part is porous with its measured properties averaged over a cross-section that is only partly filled [14]. The infill structure is of the order of the nozzle diameter in thickness, between 0.2 and 0.8 mm [14]. The orientation of the part on the build plate, the direction in which the filaments are laid within each layer, the air gap between them, the layer thickness and the degree of interlayer bonding are all fixed during printing, and together they form a mesostructure that behaves like a bonded laminate, not a bulk polymer. Any statement about the flexural strength of a printed part is thus a statement about this mesostructure, and a model that predicts it has to represent the mesostructure as well as the material.

Its most studied property is anisotropy. Because the load-bearing filaments run in a chosen direction, the stiffness and strength of the part depend on the angle between that direction and the applied load, and a large body of experimental work confirms that raster angle and build orientation are among the strongest influences on the flexural properties [16,17,18]. The two are not equivalent in size, since reorienting the whole part moves the interlayer planes relative to the span and produces the larger effect, whereas the raster angle matters most at partial infill and becomes modest once the section is solid. At full infill a raster aligned with the span carries the bending stress along continuous filaments. A transverse raster loads the weaker inter-filament bonds, so that a change of raster alone shifts both the flexural strength and the modulus [12]. In a specimen built upright the layers are stacked across the span and not along it. The weak interlayer planes are then loaded directly by the bending stress and much of the bending capacity is lost [13]. The anisotropy is also asymmetric, because the printed material responds differently in tension and in compression, so the two faces of a beam in bending are not governed by the same effective modulus [19]. That asymmetry is frequently neglected, and it follows that an isotropic description of a printed beam, which assumes one modulus for both faces, misplaces the neutral axis and misstates the bending stress.

Where the anisotropy governs how the stress is carried, the interlayer bond limits how much of it the part can take, because bending loads the interface directly. The bond between two layers forms while the newly deposited filament reheats the one beneath it, so the strength of the bond is a thermal outcome that depends on the deposition temperature, the cooling rate and the resulting degree of chain diffusion and crystallinity [20]. Where the bond is poor the part fails by delamination along the interlayer plane instead of through the filament, and fracture along the filament bonds has been characterised both by dedicated fracture tests [21,22] and by three-point bending of vertically built specimens [23]. The bond is also among the properties most responsive to intervention, since annealing a bi-material joint of PLA and nylon above the melting point of PLA raised the interfacial fracture toughness by an order of magnitude [24], a result for the interface between two polymers that is not evidence for the interlayer bond within one, and tuning the deposition parameters together locates the combination at which the flexural strength is highest [25]. Interlayer bonding therefore links the process parameters directly to the flexural response, and it is the mechanism through which most parameter effects act, yet an explicit representation of that interface remains rare in the models that predict bending [26].

Both of those properties are set in practice by the process parameters, and that parameter space has been mapped in great detail. Infill density and pattern, layer height, raster angle, build orientation, printing speed and the nozzle and bed temperatures all shape the mesostructure, and their influence on the flexural and related mechanical properties has been the subject of many experimental and optimisation studies [27,28,29,30,31,32,33]. Statistical and design-of-experiments methods such as the Taguchi array and response-surface modelling are widely used to locate the parameter combinations that maximise strength [25,33,34]. Material modification adds a further dimension, since carbon-fibre reinforcement [35], bonded composite plates [36], natural fillers and biochar [37,38] and surface treatment [39] each shift the baseline mechanical properties. The size of this literature also makes it hard to synthesise, because results are reported under different standards, geometries and conditions, so no single study shows what they have in common.

All of that work assumes planar layers, although the layering scheme is also a design variable. Planar slicing can approximate a curved surface only by a stack of flat layers, which leaves a stepped profile that becomes more pronounced as the surface flattens toward the horizontal, and the layer interfaces of such a stack increase the chance of delamination failure [40]. Non-planar and curved-layer deposition removes the steps by letting the layers follow the surface, and it further allows the toolpath to be aligned with the load so that continuous filaments, rather than weak interfaces, carry the bending stress [41,42]. The approach is not new. Curved-layer FFF has been demonstrated experimentally and supported by dedicated slicing algorithms over the past decade [43,44,45,46], and it has been extended from single curved skins to parametric lattice structures [47] and to multi-axis machines that reorient the part to print with little support [48,49] or along chosen directions [50,51]. Curved-layer deposition raises the flexural strength directly, and the increase over planar-built parts has been measured and related to the fill gap and the resulting filament geometry [52]. Some of the largest mechanical improvements reported in the field come from this class of methods, reaching well beyond 100% in bending force for a non-planar over a planar beam in individual continuous-fibre cases [41]. In unreinforced parts, a toolpath along the principal stress lines raises the strength by 50% for the same amount of material [53] and an adaptive-density infill raises the maximum flexural load by 140% [54], though the size of the effect depends on the geometry and the loading. Printing such paths requires both a slicer that can generate them [55,56] and a machine that can execute them [57]. The mechanical promise of non-planar deposition is therefore well established experimentally, while the means of predicting it numerically is not.

Finite-element prediction brings its own difficulties, because the model has to represent the anisotropy and the weak interlayer bond. A model must choose the scale at which it represents the mesostructure, from a homogenised continuum with effective properties [58,59,60], through a mesoscale or representative-volume description that resolves the filaments and voids [15,61], to an explicit interface model that places a cohesive law between the layers [26]. It must also choose the material model, and here the central question is whether the printed part is treated as isotropic or as orthotropic. A model that gives the printed geometry the properties of solid tensile specimens cannot see the weak interlayer plane and can overestimate the bending strength [62], whereas an orthotropic or homogenised description carries the anisotropy through effective properties and correlates more closely with experiment [59,61]. The reported agreement between model and bending test spans a wide band, from a few percent [59,63,64] to between 27 and 58% for such a model, which overpredicts the flexural stress [62], and this pairing of a bending experiment with a matching simulation has been pursued for a high-performance engineering polymer [65], for sandwich structures [66,67,68] and for topology-optimised parts [69,70]. In tension, a large error has been closed by calibrating the modelled raster cross-section against the printed one [71]. Process-level simulations of deposition and distortion complete the picture, since the residual state they predict sets the starting point for a later bending analysis [72,73]. What these accounts do not settle is how consistently such a prediction is checked against a bending experiment, since a model of a printed part may equally be validated against tension, against compression, or against no experiment at all.

The field has been surveyed repeatedly, yet along separate axes. Reviews of the process parameters and of property-enhancement approaches catalogue the influence of raster, infill and temperature on strength [28,74,75], reviews of the materials compare polymers, composite filaments and bicomponent prints [27,34,76], and reviews that include the numerical work collect finite-element approaches to non-uniform infill and to residual stresses [29,77]. Non-planar deposition has likewise been treated on its own terms, with planar and non-planar slicing algorithms compared for their effect on surface quality, build time, support structures and mechanical properties [78]. A recent systematic review goes further and aggregates the mechanical evidence itself, reporting across 24 studies that non-planar toolpaths raise mechanical performance by 49.2% on average while 17.2% of the experiments record a loss, and an average of 80% where the toolpath is computed from a finite-element stress field [79]. That aggregate records what has been measured, and the finite-element analysis within it serves the design of the toolpath and not the prediction of the response. Each of these covers its own part of the problem well. The parts are not independent, however, and none of the reviews identified by this search follows the deposition path through the anisotropy it creates to a prediction of the bending response that has been checked against experiment.

The present review treats three lines of work that are usually reviewed separately as consecutive steps, in which the process parameters set the mesostructure, with its anisotropy and the quality of its interlayer bond, the toolpath orients that mesostructure with respect to the load and fixes the surface geometry, and the finite-element model must represent both if its prediction of the bending behaviour is to hold against experiment. Its contribution relative to the reviews above lies in how the finite-element evidence is treated. Every cited study that builds its own finite-element model of a printed part, 84 in all, is classified by deposition strategy, by the purpose of the model and by the experiment it is compared with, and the classification is provided study by study in Supplementary Table S1. A comparison with a bending test is kept apart from one with tension, compression or no experiment, and an independent comparison from a calibrated one, and the agreement of the validated models is reported separately for the elastic stiffness, the load–deflection response, the flexural strength and damage. Because planar and non-planar deposition are read through the same classification, the shortfall in the flexural modelling of non-planar parts can be stated as a count and its cause located. It works from the flexural response and covers the material set that appears most frequently in the recent literature, namely polylactic acid (PLA), acrylonitrile butadiene styrene (ABS) and the copolyester polyethylene terephthalate glycol (PETG), with other polymers considered where a study is methodological. Section 3 examines the mesostructure through the process parameters, the anisotropy and the interlayer bond, Section 4 planar and non-planar deposition and the toolpaths each allows, and Section 5 and Section 6 the finite-element modelling of the flexural response and its validation against experiment. Section 7 examines the finite-element prediction of non-planar deposition, the step with the least numerical support.

## 2. Scope and Review Method

This is a narrative, state-of-the-art review that reads the field through the flexural response. It is neither a systematic review nor a meta-analysis. It follows a transparent and reproducible search, so that the corpus behind the quantitative summaries can be audited, but the synthesis remains interpretive and the counts reported here describe the reviewed set and not the whole field.

### 2.1. Scope and Research Questions

The review asks how the mesostructure, and the toolpath that orients it with respect to the load, govern the flexural response, and how far finite-element models predict that response when they are checked against a bending experiment. The emphasis is on peer-reviewed journal and conference articles published between 2015 and 2026, with the weight of the recent evidence drawn from 2021 onwards. Work published before 2015 was admitted only where later studies build on it, which was taken to mean that at least two of the studies from 2015 onwards read here, or of the reviews used as seeds for backward snowballing, cite it, and the four such studies cited concern bond formation between filaments, the processing conditions of that bond, the anisotropy of printed ABS and process-level finite-element simulation. The material focus rests on PLA, PETG and ABS, with other polymers and their composites retained where a study is methodological and its findings do not depend on the material. Metal additive manufacturing, powder-bed fusion, vat photopolymerisation, bioprinting and purely thermal process simulations lie outside the scope.

### 2.2. Literature Search and Screening

The literature was searched in Scopus, in Web of Science and in the OpenAlex bibliographic index with the five queries listed below, applied to the title and abstract, on Web of Science through the topic field. The records screened were drawn from OpenAlex, which returned the most records on S1 and S5, the two queries that carry the central claim, and the Scopus and Web of Science returns were checked against them (Table 1). Every record was verified against Crossref, which established the digital object identifier that the eligibility rule requires, and the full texts were obtained from the publishers’ platforms.

The strategy combined six concept groups of keywords, namely deposition strategy (non-planar, nonplanar, curved-layer, multi-axis), process (fused deposition modelling, FDM, fused filament fabrication, FFF, material extrusion), flexural response (flexural, bending, three-point bending), process parameters (process parameter, layer thickness, raster, infill), interlayer bonding (interlayer, delamination, interfacial bonding) and numerical modelling (finite element, cohesive zone, phase field, representative volume element). These groups were assembled into 5 queries.

**S1**  (non-planar OR nonplanar OR curved-layer) AND (FDM OR FFF OR fused deposition OR material extrusion)
**S2**  (flexural OR bending OR three-point bending) AND (FDM OR FFF OR fused filament) AND (process parameter OR layer thickness OR raster OR infill)
**S3**  (interlayer OR inter-layer OR delamination OR interfacial bonding) AND (FDM OR FFF)
**S4**  (finite element OR cohesive zone OR phase field OR representative volume element) AND (FDM OR FFF OR fused deposition)
**S5**  S1 AND (flexural OR bending OR mechanical properties)

The following eligibility filters were applied.

- Publication period from 2015 to 2026, with earlier work retained only where it is foundational.
- Peer-reviewed journal or conference articles carrying a digital object identifier.
- English language.
- Polymer material-extrusion (FDM or FFF) subject matter.

After de-duplication by record identifier and digital object identifier the database search identified 3498 records, and backward snowballing from 14 seed reviews added 788. Removing, automatically from the record type and the identifier, 733 non-article records, namely preprints without peer review, theses, datasets and book chapters, left 3553 article records, which were screened on title and abstract. A study was retained when it reported experimental flexural data, described a planar or non-planar deposition strategy, or presented a numerical model of the mechanical response, and it was set aside when it addressed a purely thermal process with no mechanical outcome or fell outside the scope. Every record carried a relevance score, computed automatically from its title and abstract with a fixed set of keyword terms, which set the order in which the records were considered and not their eligibility. The score adds 30 points for each of the three lines of the review that the record addresses, namely deposition strategy and toolpaths, process parameters and flexure, and numerical modelling, 25 for a match to the intersection query S5, 20 for a flexural or bending term, 15 for a numerical model combined with an experiment and a mechanical outcome, 12 for a non-planar or curved-layer term, 10 for a named focus polymer, 6 for a delamination or interlayer term, up to 15 for the number of citations per year and 5 for publication from 2024 onwards. It subtracts 25 for a record that names another additive process, 20 for one that matches none of the themes, 12 for one without a mechanical outcome and 6 for one whose only named materials lie outside the focus, with a floor of zero. The material term listed PLA, PETG and acrylonitrile styrene acrylate, the polymers of the initial scope, so the ABS studies that entered the scope later were ranked through the other terms. Records scoring at least 90, together with every record on deposition strategy, formed a list of 454 candidates for full-text reading. The cut-off of 90 was a working value set to give a candidate list that could be read in full, and it was not derived from a calibration. It did not apply to records on deposition strategy, which were candidates whatever their score, so the non-planar side of the central count does not depend on it. The score did not decide inclusion either, since 72 of the 139 included studies that come from the screened records score below 90, and 48 of them were not on the candidate list. Of the 23 flexural validations among the screened records, 22 score at least 90, and the one that scores lower, at 75, was read although it was not on the candidate list, so no validation found lies just below the cut-off. Eligibility was decided on the full text against the criteria above. Full texts were obtained in successive rounds in order of priority and score, first those openly available, then through institutional access and, where neither gave access, by request to the authors, which provided one study. Acquisition stopped when the included set approached the intended scope of the review and the disconfirming search described below had shown that further records did not change the central count, and candidates not obtained by then were not read. This gave 192 papers read in full, of which 170 are included in this review. Every cited primary study that builds its own finite-element model of a printed part was classified by deposition strategy, by the purpose of the model and by the experiment it is compared with, which gave a set of 84 studies, and the one further such study added by the fifth check described below is discussed in the text but not added to that set [80]. The intersection query S5 matched 64 records, 47 of them articles, and a return of that size is not taken as evidence of a gap, because it depends on the vocabulary the authors of the studies chose.

Five further checks followed, and their records are kept apart from the main counts. A deepening search of 22 queries on curved-layer, conformal, multi-axis and robotic deposition returned 53 candidates not already held, of which 29 were added. A disconfirming search of 12 queries sought studies combining non-planar deposition with a finite-element model and an experimental bending validation, and of 594 records examined it added one, a non-planar model that on full reading is validated in tension and not in bending [81]. The 41 records that the initial screening had set aside by a stricter cut-off of 2021 were re-examined, and 3 were added, one of them a validated finite-element bending study. Finally, every record that S5 returned on Scopus or Web of Science was assessed and either cited or excluded, and the 164 records with a digital object identifier that S1 returned on the two services were checked against the cited set, which together added four studies [79,82,83,84]. A fifth check extended the treatment of polymer properties with four queries, on the thermal state of the polymer, on moisture and ageing, on the rate and temperature of the test, and on finite-element bending models that are not purely elastic, the last built to break the finding that the reviewed models treat the polymer as elastic. Of the 467 articles returned by the open index and the 81 further records found only on Scopus or Web of Science, 62 were referred to full text on title and abstract, 28 of these were obtained and read, and 19 met the eligibility rule, of which the 17 that add a distinct finding are cited, while none of the 28 describes the polymer in a finite-element bending model as viscoelastic. The deepening search, the disconfirming search and the record-by-record check were built around non-planar deposition, and no search of comparable depth was run for planar finite-element studies, although query S4 alone returned between 1373 and 1972 records depending on the database (Table 1). The planar finite-element set is therefore a lower bound, and the planar and non-planar shares compared in this review come from two samples searched with different effort.

Planar deposition denotes flat-layer fabrication, and curved-layer or non-planar deposition denotes fabrication in which the layers or the toolpath leave the build plane and follow a curved surface or a load path, whether on a three-axis machine or with more than three controlled axes, the latter being multi-axis material extrusion. Curved-layer and non-planar deposition are treated as one category in every count of this review, and where a statement applies only to layers that follow a single curved surface on a three-axis machine, which is the form of the one validated flexural case, it says so. Continuous-fibre extrusion is treated as a distinct material system and not as evidence for unreinforced deposition. A finite-element bending study is one whose prediction is compared quantitatively against a three or four-point bending or an equivalent flexural experiment, such as a cantilever bending test, and validation denotes that comparison. A validation is calibrated when the interfacial or cross-sectional properties of the model were fitted to tests on the same material system, or when its material law was taken from the very tests it is compared with, and independent otherwise. Errors between model and experiment are relative to the measured value and keep their sign, so that overprediction and underprediction are distinguished. The study counts are counts of published works, whereas each plotted flexural value is one reported specimen condition.

Each classification decision was recorded with the experiment, the page and a verbatim quotation from the study, and the complete record was then checked by two further authors. They worked on a form that stated the eligibility rule and the definitions above and listed all 84 decisions with their quotations, the borderline cases first, together with the 16 cited studies that use finite elements without building their own model of a printed part and were therefore kept out of the set, and they had the full text of every study. For each decision the checker recorded agreement, or the correct decision with a reason. The second check was made on the form completed in the first, so the two checks were not independent and no chance-corrected agreement statistic is reported. The first checker agreed with 82 of the 84 decisions and the second with 81, a raw agreement of 98 and 96% that overstates independent agreement, and both confirmed all 16 exclusions. The 3 disputed decisions and further corrections recorded in the comments were verified in the source, and once each correction had been verified in the source, 9 entries were changed. One study entered the flexural set, one left it, one validation was reclassified from independent to calibrated, a numerical deviation was added for three studies and its wording was corrected for three others. The complete classification, with the experiment, the quotation and the reason for each exclusion, is given in Supplementary Table S1.

## 3. Process Parameters, Anisotropy and Interlayer Bonding in Flexure

**Figure 1:**
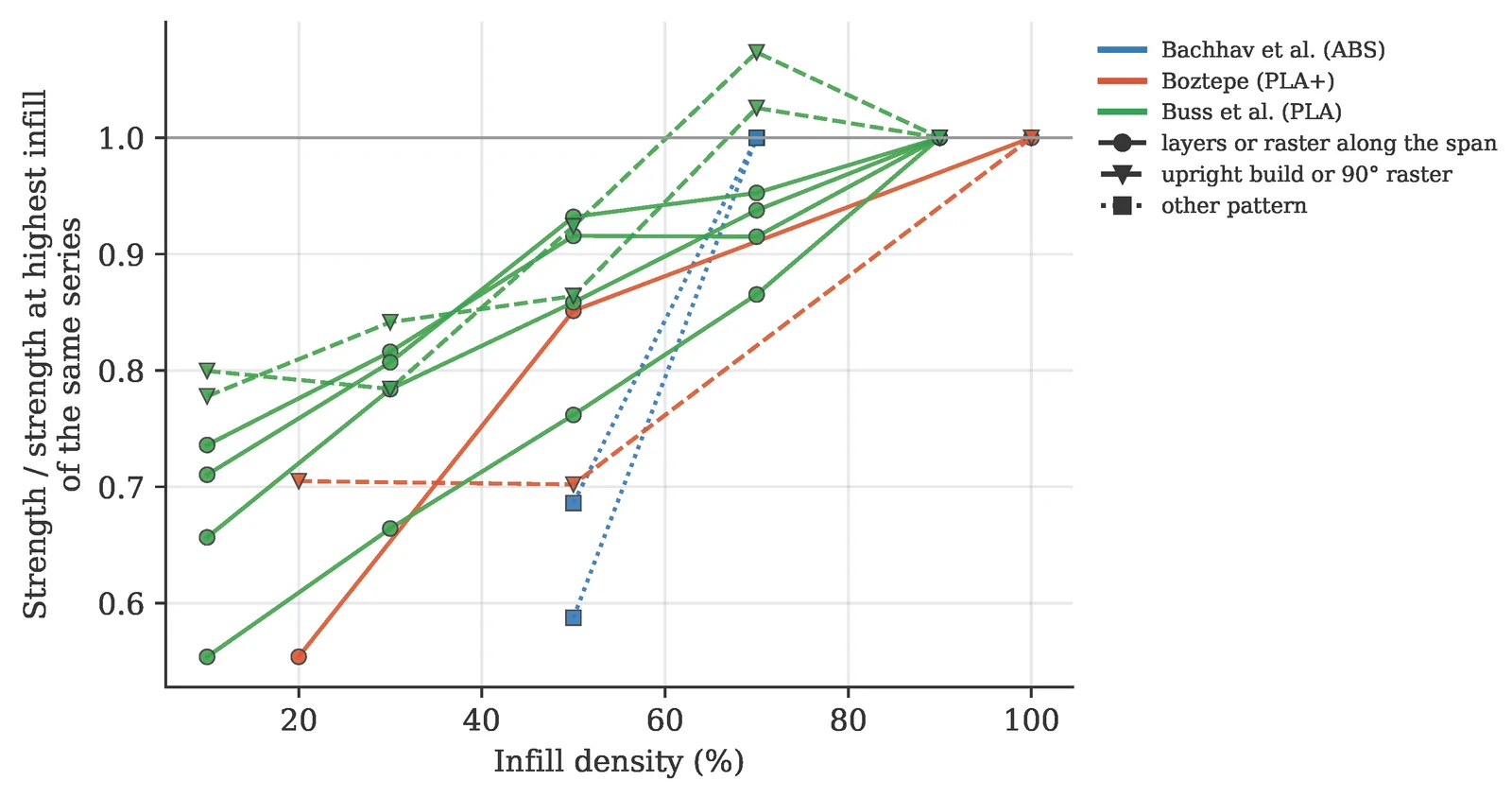
Flexural strength relative to the highest-infill condition of the same study, material, orientation and pattern, for the ten series of neat focus polymers that report at least two infill levels, PLA+ from [12], PLA from [13] and ABS from [62]. The horizontal line marks a ratio of 1.

Infill density has a strong influence on flexural strength, particularly at low to moderate relative density, but its effect is conditional on the shell architecture, the raster orientation, the build orientation and the specimen geometry, and it is not dominant in every case. With a zero-degree raster the flexural strength reaches a maximum of 101.82 MPa at full infill, against 86.68 MPa at 50% and 56.40 MPa at 20%, so the increase is strongly non-linear and most of it occurs between 20% and 50% [12], while in another series the flexural strength saturates, with a clear plateau beyond roughly 50 to 60% fill, above which the failure becomes shell-dominated [13]. The raster pattern modifies this baseline. A cubic pattern outperforms a triangular one at every density in the two horizontal builds, while in the upright build the triangular pattern is the stronger [13], and the dependence of the elastic properties on the infill pattern has also been captured numerically [85]. Material choice scales the whole picture, with flexural strengths of 90, 85 and 80 MPa measured for PLA, PETG and ABS at full density [70]. How build orientation, layer thickness and feed rate act on both the tensile and the flexural response of PLA, and which parameter combinations optimise them, has been mapped systematically [86]. Infill and raster are prominent among the parameters studied for the strength of printed parts, in bending as in tension and fracture, across a wide range of materials and machines [30,31,32,87,88,89,90], without being universally dominant, and in multi-material prints the combination of filaments matters as well, since delamination between them lowers the flexural strength [91]. Figure 1 shows the effect of infill within each study, with every strength divided by that of the highest infill in the same series, a series being one study, material, orientation and pattern. Ten series from three studies report at least two infill levels for a neat focus polymer [12,13,62]. Normalised in this way, the strength at the lowest reported infill lies between 0.55 and 0.80 of the highest-infill value. The reduction is smaller where the interlayer planes or the filaments cross the span, and in both upright series of one study the strength at 70% infill slightly exceeds that at 90% [13]. Absolute values are not pooled across studies in this figure, because they come from different standards, specimen geometries and grades and would be read as a ranking of the polymers that the data do not support.

**Figure 2:**
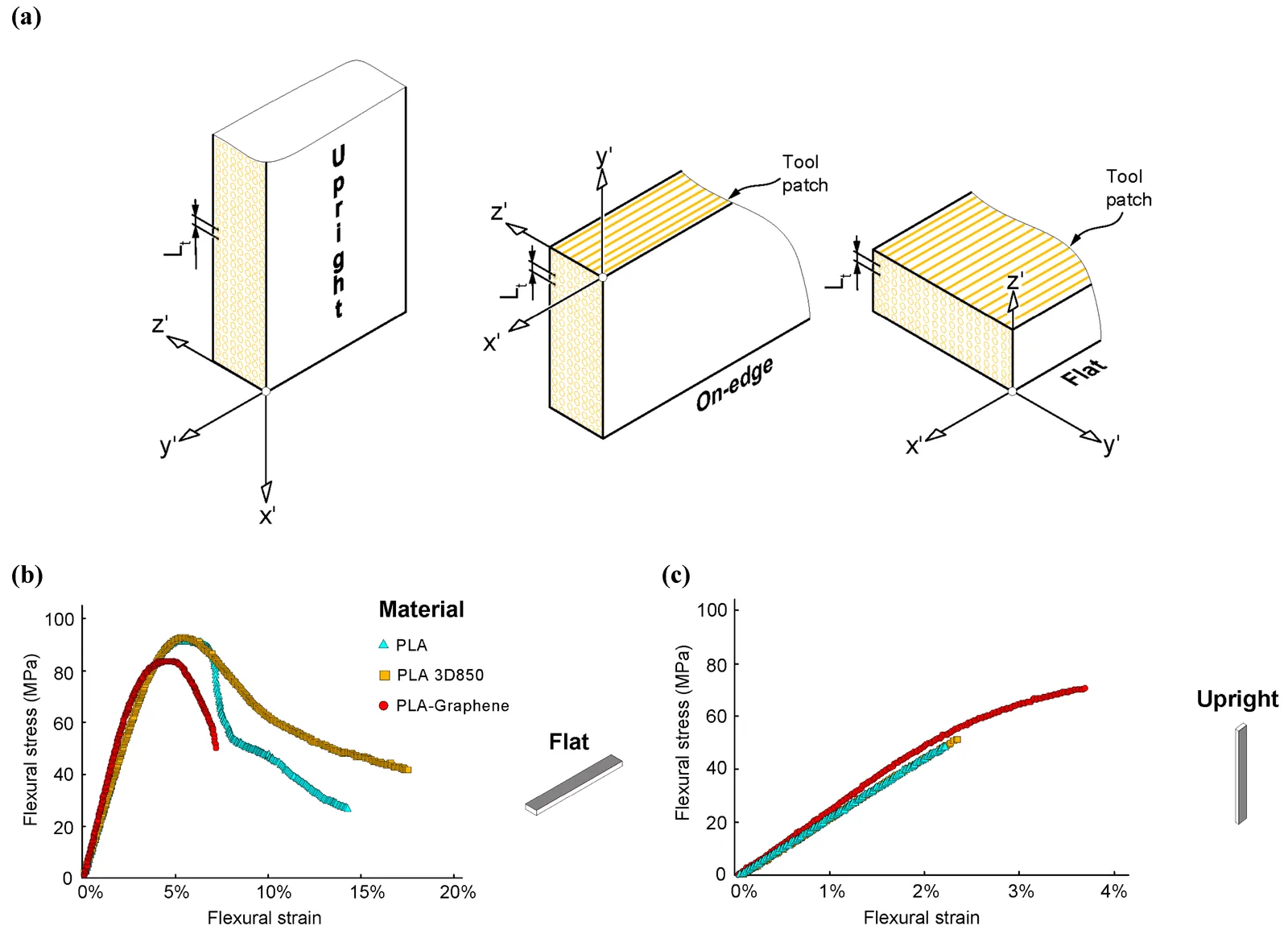
Upright, on-edge and flat build orientations (**a**) and average flexural stress–strain curves of three PLA-based filaments printed flat (**b**) and upright (**c**). Adapted from [93], MDPI, 2019.

**Figure 3:**
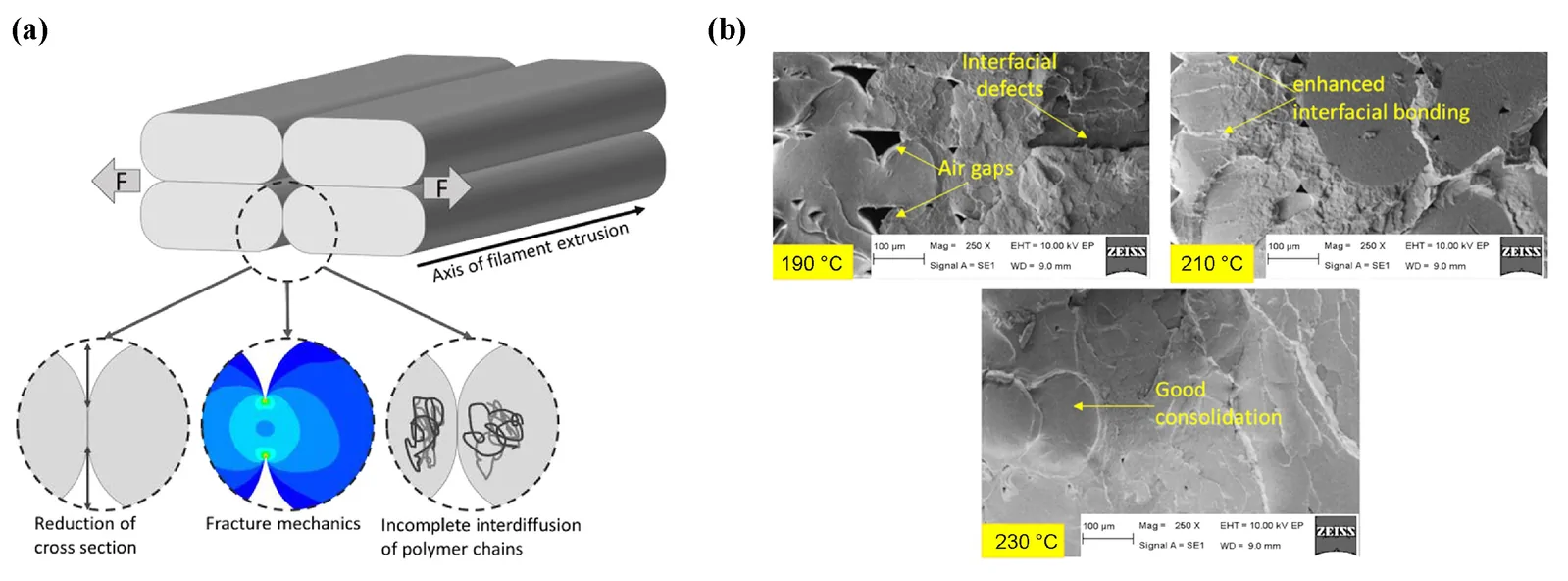
Strength-reducing mechanisms under loading transverse to the filaments (**a**) and tensile fracture surfaces of PLA printed at extrusion temperatures of 190, 210 and 230 °C (**b**). In (**a**), F denotes the applied force. Reproduced from [95], Springer Nature, 2019, and [98], Springer Nature, 2024.

The direction of the filaments relative to the load is as important as their amount, and this dependence was established early, when the anisotropic properties of fused deposition ABS were characterised and related to the raster orientation and the air gap between filaments [92]. The later raster studies build on that measurement. The two variables act on different scales, and separating them matters. At full infill the raster angle is a secondary effect, since a zero-degree raster gives only a 6.9% higher flexural strength and a 14% higher modulus than a ninety-degree one once the section is solid [12], while at partial infill the same study shows the raster changing the strength substantially, from 56.40 to 67.13 MPa at 20% depending on the angle. Build orientation has the larger effect. The flat, on-edge and upright builds differ in how the layers meet the span (Figure 2a). In the flat and on-edge builds the layers run along the beam, whereas in the upright build every layer interface crosses it, and PLA-based specimens printed upright fail at a lower flexural stress and a much smaller strain than the same materials printed flat (Figure 2b,c) [93]. The upright build is the weakest for both infill patterns at every density, between 10.01 and 16.66 MPa [13], and at 90% cubic infill the flat build reaches 30.73 MPa against 12.87 MPa upright, a factor of more than two. The factor shrinks at low density, where the weakest horizontal build, flat with a 10% triangular infill, reaches 14.43 MPa against 12.41 MPa upright. The same anisotropy governs durability, with the longest fatigue life at a thirty-degree raster and the shortest at ninety degrees [94]. Because the anisotropy originates in the deposited mesostructure and not in the polymer, computed tomography has been proposed as a route to characterising it directly and non-destructively [95]. The same work names three mechanisms by which the deposited mesostructure weakens a part loaded across its filaments, namely the loss of cross-section to voids, the stress concentration that the voids create and the incomplete interdiffusion of polymer chains across the interface (Figure 3a).

The upright build is the weakest because its bending stress crosses the interlayer planes, where the strength of the bond between the layers limits the flexural strength. The underlying mechanism is a sintering sequence in which two deposited filaments first come into contact, then form a neck that grows while the material remains above its transition temperature, and finally develop strength through molecular diffusion across the interface. Models of this bond formation for extruded filaments show that it is governed by the cooling profile of the deposited material, with sintering and molecular diffusion controlling the resulting bond [96], and the processing conditions, in particular the envelope temperature and the cooling history, determine the mesostructure and the bond quality that follow from them [97]. A cohesive law at a printed interface represents the outcome of this process, so its parameters hold only for the thermal history under which they were measured. That bond is a thermal outcome. Measured by atomic force microscopy, the local elastic modulus is 220 MPa in the topmost part of a specimen, farthest from the heated build plate, where the authors detect poor adhesion between the layers, against 500 MPa in its central part, and every specimen stayed below 3% crystallinity [20]. The extrusion temperature acts differently, with the flexural strength of PLA falling from 59.06 to 50.53 MPa as the extrusion temperature rises from 190 to 230 °C [98]. Over the same range the tensile strength of that PLA rises from 43.3 to 68.3 MPa, and its tensile fracture surfaces change from air gaps and interfacial defects at 190 °C to a consolidated interface at 230 °C (Figure 3b). A better interlayer bond therefore raised the tensile strength while the flexural strength fell, and the authors discuss the effect of the extrusion temperature in terms of molecular alignment and crystallinity in the polymer. The finding rests on a single study, but it shows that the flexural response of a printed polymer is not set by the interlayer bond alone. The largest single improvement reported for an interface comes from post-processing, since annealing above the melting point of PLA raises the interfacial fracture toughness of a bi-material joint of PLA and nylon by up to 1225% [24], a result that again concerns two polymers and not the bond within one. When the parameters are tuned together the bond can be optimised directly, and a flexural strength of 78.52 MPa has been reached at a layer height of 0.1 mm, a speed of 60 mm/s and a temperature of 200 °C [25]. The same weakness of the bonds between deposited filaments controls fracture, which has been assessed through the essential work of fracture of specimens printed with different raster angles [21], through notched-specimen analysis [22] and through dedicated characterisation of the interlayer shear strength [99], and printed structural joints have been assessed in tension and three-point bending, both bonded with an adhesive [100] and interlocking [101]. Printing temperature and thermal history enter here as well [102].

The polymer takes part in this bond through the state of its chains, and the studies disagree on which state governs bending. Where the crystallinity stays below 3%, as in the study above, the interlayer performance was referred to the chain entanglements rebuilt by diffusion across the interface and not to crystals [20]. In another PLA, raising the nozzle temperature from 210 to 240 °C increased the crystallinity from 7.48 to 17.39% and the flexural strength of specimens whose deposited lines run perpendicular to the loading direction from 52.94 to 62.22 MPa, while specimens with the lines along it stayed between 66.58 and 67.78 MPa [103]. This rise runs opposite to the fall in flexural strength with extrusion temperature described above [98], and because the two studies differ in grade, temperature window and build orientation, the direction of the effect remains open. Crystallinity and flexural strength can also move apart, since PLA printed at full infill at an ambient relative humidity of 80% instead of 25% lost 20% of its specific flexural strength while its crystallinity rose, which the authors attribute to hydrolytic scission of the chains [104]. Post-print heat treatment depends on the same thermal window. Annealing below the melting point lowered the mixed-mode fracture toughness of a PLA and nylon interface by 88% [24], whereas PLA annealed at 60 °C for 5 h reached a flexural modulus of 2337.73 MPa and a flexural strength of 63.96 MPa, against 2164.40 MPa and 59.66 MPa without annealing [105], and PLA annealed at 132 °C reached a flexural strength of 117.7 MPa against 103.0 MPa after annealing at 63 °C [106]. ABS annealed between 100 and 135 °C in the same study stayed between 65.1 and 67.2 MPa [106], and the flexural modulus of amorphous PETG rose by 4.6% after annealing at 100 °C and fell by 20.8% after annealing at 190 °C (computed here) [107]. Where the geometry is held during annealing the improvement can be large, and an ABS core annealed inside a printed polycarbonate shell raised its fracture toughness in single-edge notched bending eighteenfold, from 0.285 to 5.14 kJ/m^2^ [108]. Heating the previous layer with a laser during deposition raised the peak interface temperature of ABS from about 77 to about 140 °C and its peak flexural strength from about 18 to about 45 MPa, before surface damage lowered the strength at the highest laser power (strength values read here from the plotted data) [109]. The same temperature can strengthen one polymer and destroy another, since holding printed ABS with filaments along the specimen at 100 °C raised its plane-strain fracture toughness by 88.26% while PLA held at that temperature lost toughness and deformed beyond testing within 30 days [110]. A thermal trend measured on one filament therefore does not transfer to another, and its sign follows where the treatment temperature lies relative to the glass transition and melting points of that polymer.

All of these parameter effects are measured on a particular polymer, and the choice of polymer moves the baseline on which they act. For the PLA, PETG and ABS that are among the most frequently studied in this literature, the ranking depends on the property. One study places PLA highest in flexural strength at 90 MPa [70]. Another finds PETG strongest in bending at 70.62 MPa but PLA stiffer, at a flexural modulus of 2.07 GPa [111]. The two results are not directly comparable, since they differ in material grade, specimen design, processing and test conditions, and the apparent disagreement most likely reflects those differences, and no fixed ranking of the polymers follows from it. Reinforcement, fillers and surface treatment shift these baselines further, in carbon-fibre PETG [35], PLA reinforced with graphene nanoplatelets [93,112], biochar-filled [37] and natural-fibre biocomposite [38] filaments, in PLA parts strengthened by bonded carbon-fibre composite plates [36] and in PETG after a cold-plasma treatment of each layer [39], whereas seawater ageing has been assessed through microhardness and not through a flexural test [113]. These shifts are consistent with the wider literature on fibre-reinforced material extrusion [114] and with the role of the processing parameters in setting the resulting microstructure [115]. Where the reinforcement lies in the architecture of the part, the flexural response follows the same logic, as in short-carbon-fibre sandwich panels [66] and in structures reinforced with cellular and fractal patterns [116]. Table 2 compiles measured values as reported, separately for the neat focus polymers and for reinforced or blended materials, in which CF denotes a carbon-fibre reinforcement and n.r. a value not reported. The values were obtained under different standards, specimen geometries and printing conditions, so they show the range of each material and cannot be used to rank them.

The two extreme PLA-based entries in Table 2 differ by a factor of more than three, at 101.82 MPa [12] and 30.73 MPa [13], and the reported conditions suggest that little of the difference comes from the test standard itself. The higher value is a modified commercial grade tested at full infill with the raster aligned along the span, so continuous filaments carry the tensile fibre stress, and it was measured to ASTM D790 over a 51.2 mm span. The lower value is an unmodified PLA at 90% cubic infill, where a three-dimensional cell architecture carries the load, measured to ISO 178 over a 64 mm span. Material grade and load-path architecture therefore account for most of the gap, with the standard and the span contributing the smaller part, which is consistent with the mesostructure rather than the polymer setting the flexural response. It also means that a single quoted flexural strength for a printed polymer carries little information without the raster, the infill architecture and the grade alongside it.

**Figure 4:**
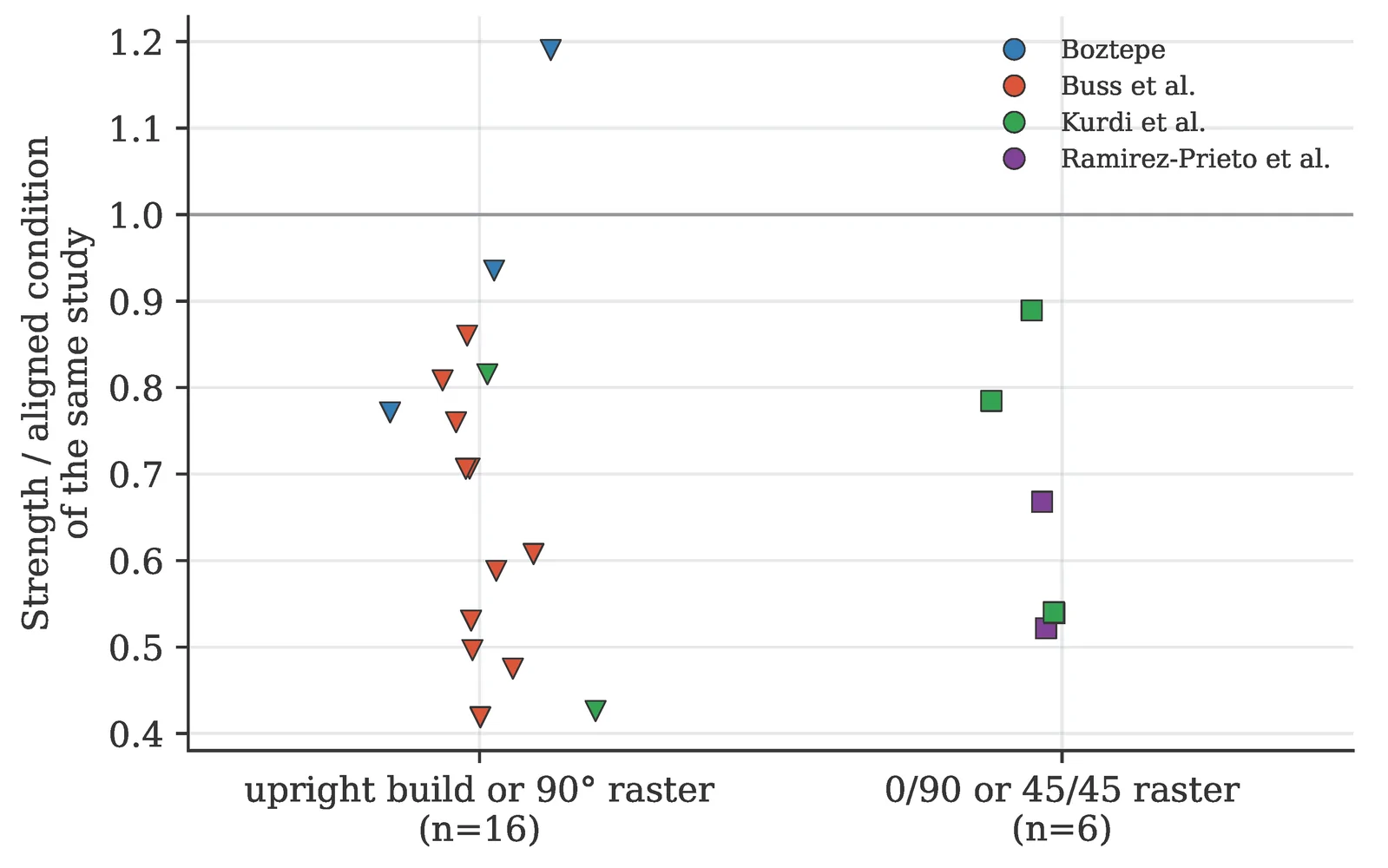
Flexural strength of upright, ninety-degree and cross-ply conditions relative to the aligned condition of the same study with the same infill, pattern and specimen thickness, for PLA [13,16] and PLA+ [12,17]. Triangles mark upright or ninety-degree conditions and squares cross-ply conditions.

Figure 4 isolates the orientation in the same way. Each upright, ninety-degree or cross-ply condition is divided by the aligned condition of the same study with the same infill, pattern and specimen thickness, which is the flat build where the build orientation varies and the zero-degree raster where the raster does. Four studies report such pairs for a neat focus polymer, giving 22 ratios [12,13,16,17]. Of the 16 upright or ninety-degree conditions, 15 lie below their aligned reference, at 0.42 to 0.94 of it, and the one exception is a ninety-degree raster at 20% infill, which reaches 1.19 times the zero-degree value [12]. The six 0/90 and 45/45 conditions lie at 0.52 to 0.89. The orientation effect therefore appears within every study that reports it, and its size depends on the build, the infill and the specimen, which a comparison of absolute strengths across studies cannot show. The absolute values are listed in Table 2 and are not pooled into medians, because each pooled group would mix infill densities, orientations, grades and test standards.

## 4. Planar and Non-Planar Toolpaths and the Staircase Effect

**Figure 5:**
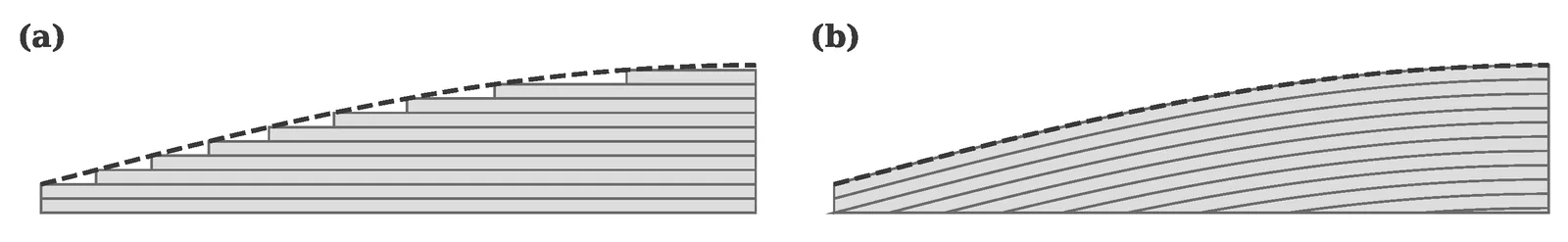
Schematic of a rising curved surface produced with (**a**) planar layers, which approximate it in steps, and (**b**) layers that follow it.

On a curved surface a planar slicer can only approximate the true contour by a stack of flat layers, which leaves a stepped profile, as Figure 5 shows. The stair-stepping becomes more pronounced, with a greater distance between the steps, as the surface angle decreases toward the top of a curved print, and the layer interfaces increase the chance of delamination failure [40]. Non-planar and curved-layer deposition removes the steps by letting the layers follow the surface, and it has been measured to reduce the surface roughness by 63% relative to a planar-sliced counterpart [41]. Lattices fabricated and tested with curved layers alongside planar ones make the two strategies directly comparable under three-point bending [42]. Non-planar deposition has separately been demonstrated at large scale [119] and pursued specifically to improve surface quality [120], and its parts have since been characterised under impact loading [121], while deposition onto uneven and inclined surfaces has been controlled through a measurement of the interlayer contact force [122].

**Figure 6:**
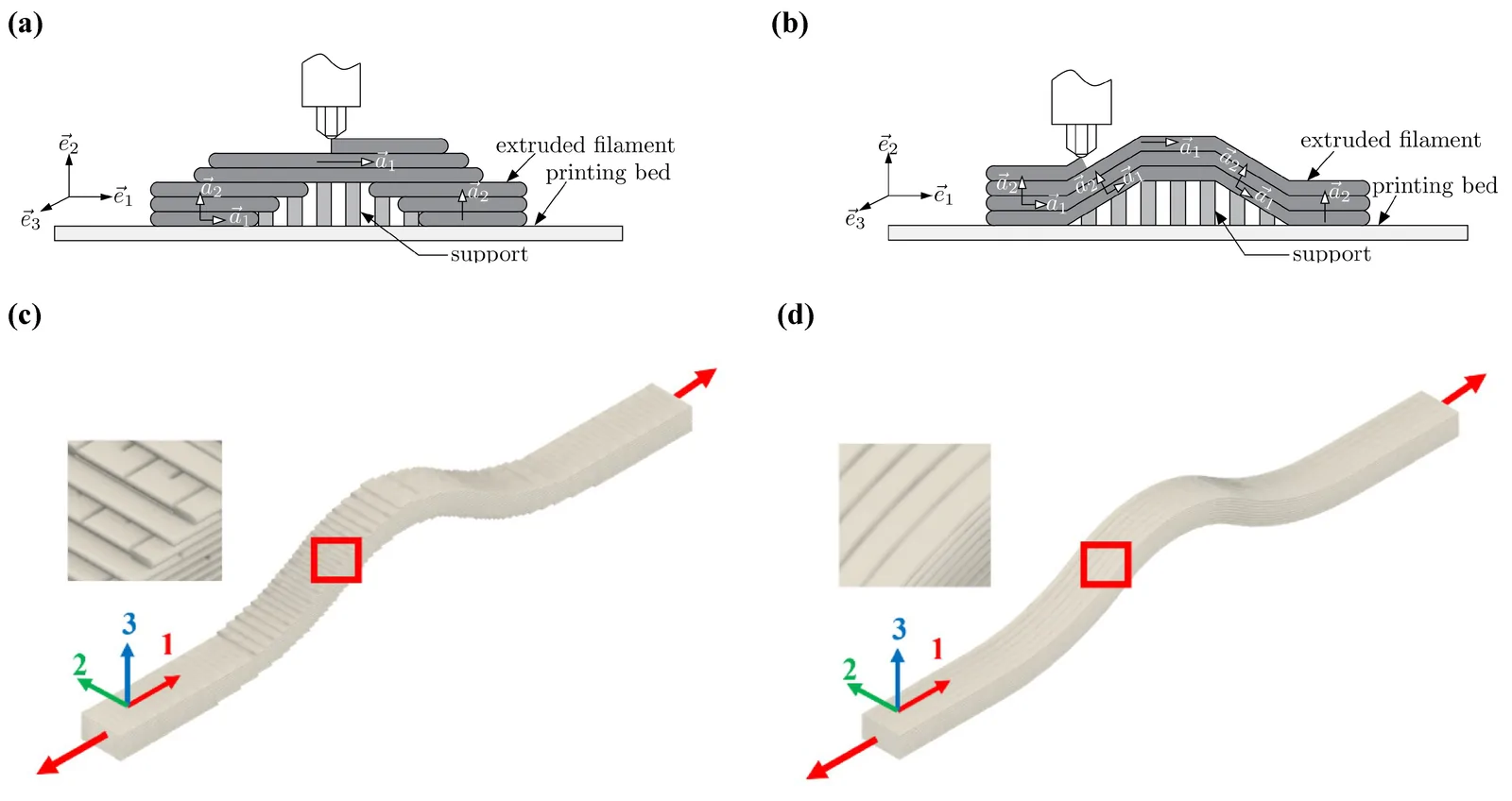
Printing direction ${\vec{a}}_{1}$ and building direction ${\vec{a}}_{2}$ in planar (**a**) and non-planar (**b**) printing and a curved short-carbon-fibre PLA bar with an XY-planar (**c**) and a curvature-following multi-axis toolpath (**d**). Red arrows indicate the direction of the applied load. Adapted from [81], Springer Nature, 2026, and [126], Springer Nature, 2025.

The surface is only the visible side of the toolpath. Beyond it, the toolpath sets the direction of the load-bearing filaments, and under bending the filaments that run along the span carry the tensile fibre stress. The toolpath also fixes the material axes of the part. In a planar print the building direction is vertical everywhere, whereas in a non-planar print it turns with the layer, so the anisotropy follows the deposition path (Figure 6a,b) [81]. How strongly this matters shows in lattices with diagonal rasters, which are about 70% softer than those aligned with the principal axes [42]. Two opposite strategies follow. One aligns the path with the load, as in principal-stress-line slicing, where the infill toolpath is generated along the principal stress lines instead of as a uniform infill pattern [53]. The other deliberately randomises the path, as in an isotropy toolpath that distributes the path directions randomly within each layer and raises the tensile strength by at least 20% over conventional toolpaths [123]. On curved surfaces the corresponding variable is the filament orientation relative to the local slope, which has been studied for non-planar thin shells and optimised for load-bearing capacity and surface quality [124]. At multi-material interfaces, interlacing the infill of adjacent materials through layered depth material images raises the interfacial bond [125].

The size of these effects depends on how much freedom the path is given. When it is allowed to leave the build plane and follow the load, several studies report large improvements in mechanical performance, although they are measured against different tests and are not directly comparable. For a continuous-fibre beam the maximum bending force rises by more than 170% over a planar equivalent [41]. Non-planar curved beams in carbon-reinforced PEEK reach an effective bending stiffness about 28% higher than their planar counterparts, alongside a 31% smaller surface deviation [82]. The largest headline numbers come from continuous-fibre systems, where the path is also the fibre path, as in a multi-axis continuous-carbon-fibre part that reaches a fibre-direction tensile strength of 190.76 MPa against 60.31 MPa for plain PLA [126]; this is a tensile comparison between two material systems rather than evidence that the toolpath alone improves the flexural response. The same study isolates the toolpath with a single material. For a curved bar of short-carbon-fibre PLA, the multi-axis path that follows the curvature raises the maximum tensile load from 550.92 to 780.00 N over an XY-planar path (Figure 6c,d), again in tension and not in bending. The largest reported effect of all comes from a five-axis system that aligns the trajectory with the stress flow in a liquid-crystal polymer bracket, with the failure strength and the stiffness improved by factors of 44 and 6 over conventional printing [83]. That polymer is strongly anisotropic and the specimen is a structural bracket rather than a standard coupon, so the figure records what path alignment can achieve in a favourable material system and is not a flexural result comparable with the others. The same study prints isotropic PLA on both machines and finds the five-axis prototype giving the lower failure force of the two, which attributes the improvement to the deposition path and the material rather than to the hardware. Other reports concern impact [40] and the modelled stress field [127], and in shear two nature-inspired interlaced toolpath schemes in PLA give strengths 31.9% and 67.5% higher than a planar toolpath [128]. The mechanism is not limited to curved skins, since multi-axis deposition has also been used to reinforce surfaces directly [129], and non-planar toolpaths have been proposed specifically to raise mechanical performance in extrusion additive manufacturing [130].

The improvements are nevertheless conditional, and the counter-examples are as informative as the positive results. Non-planar printing raises the impact strength by 50.6% for specimens printed in their weak orientation but brings no statistically significant change for the strong orientation [121], and a stress-aligned slicing that lowers the modelled von Mises stress by up to 31% against planar slicing outperforms the competing Neural Slicer in most, but not all, of the cases tested [127]. The same dependence on the angle appears in tension for PLA [84]. Non-planar layers inclined at 10° raise the strength by 11% for coupons printed flat and by 24% for coupons printed upright, whose planar layers lie across the load, but at 30° the flat coupons are 21% weaker than their planar counterparts and the upright ones only 5% stronger, values computed here from the tabulated strengths, a difference smaller than the 8% scatter of the planar set. The clearest reversal comes from dome-shaped thin shells fabricated with both planar and curved layers, where the planar domes are the stiffer, by 16% for solid shells 4 mm thick and 24% at 2 mm, with different deformation modes for the two strategies [131]. The same pattern holds across the wider set, since a systematic review of 24 non-planar studies, 794 samples and 233 experimental sets reports a mean improvement of 49.2% and a median of 33.3%, with 17.2% of the experiments returning a lower performance than their planar baseline [79]. Non-planar deposition therefore does not always improve the mechanical response, and the direction of the change depends on the geometry, the loading and the property considered. Table 3 collects these reports grouped by the mechanical test, so that unlike metrics are not compared directly, and each value is relative to the baseline stated by its own study and is not normalised across studies. In the table, Neat denotes an unreinforced thermoplastic. Both flexural entries come from fibre-reinforced systems, a continuous carbon fibre [41] and a carbon-reinforced PEEK [82], and the largest effect overall from a liquid-crystal polymer [83], so the evidence for unreinforced polymers lies in tension, impact, shear, compression and structural tests, and none of the tabulated reports is a flexural coupon test of an unreinforced non-planar part. The adaptive-density infill is listed in a group of its own, because it is the closest planar counterpart to load-aligned deposition and shows what redistributing material alone achieves, and it is not evidence for non-planar deposition. These reports sit within the broader set of property-enhancement strategies surveyed for the process [75].

None of these paths can be printed without matching planning and hardware. Load-oriented paths in non-planar geometry have been formulated by following the maximum principal stress field [55], executed with a print head that varies the layer height dynamically [57], and extended to continuous carbon fibre [132]. Moving beyond three axes changes what the deposition can reach, and the workpiece and machine design this requires has been set out for multi-axis extrusion [49,133] and implemented as robotic systems [134], on which adaptive non-planar slicing has been evaluated for free-form parts [135]. Related planning ideas appear as adaptive-density infill, which grades the fill along a variable cross-section and raises the maximum flexural load by 140% over a uniform fill [54], and as functionally graded gyroid infill for stiffness-to-mass optimisation [136].

## 5. Finite-Element Modelling of the Flexural Response

Predicting the flexural effects of the mesostructure and the toolpath falls to finite-element analysis (FEA), and the models differ mainly in the scale at which the printed mesostructure is represented, a choice that controls how anisotropy and interlayer weakness enter the prediction. The same few modelling approaches recur across coupon, lattice and full-structure studies. The studies differ mainly in the object modelled and in the experiment it is compared with. Lattice and cellular structures are modelled mostly under compression [137,138,139,140,141], and structural or designed parts, such as infill layouts, bushings, wing structures and fatigue specimens, mostly without a quantitative comparison with a static bending test [5,142,143,144,145]. Coupons and sandwich panels are compared in bending, tension or compression [14,19,146,147]. Homogenised descriptions of the printed material [148,149,150], a cohesive law at a bi-material interface [151], time-dependent creep models [152,153] and a simulation of first-layer warping [154] complete the set, and each study is classified, with the experiment it is compared with, in Supplementary Table S1. A recent example that spans the modelling and its validation is the topology optimisation of a gyroid cantilever beam, whose numerical prediction is confirmed against strain-gauge measurements on the printed beam, while an analytical direct-integration solution that ignores the stress concentrations of the lattice departs substantially from it and is judged unsuitable by the authors [155].

Table 4 summarises how the reviewed models are actually built, from the finite elements used to the treatment of the interlayer, in which RVE denotes a representative volume element and n.r. a value not reported. The table describes how the models are built and not how well they agree, and only some of its studies are among the flexural validations, so it cannot by itself rank the influence of the modelling choices. Because the layered part is anisotropic, an isotropic idealisation cannot see the weak interlayer plane and can overestimate the bending strength [62], whereas an orthotropic or homogenised description carries the anisotropy through effective properties. Whether it therefore agrees more closely with a bending test is not settled by the validated set. Grouped by the material model, the reported errors give no clear advantage to either class at the present sample size, since the two largest deviations come from isotropic models [62,156] while orthotropic and homogenised models also depart by up to 30% [7,58]. Explicit delamination, represented by a cohesive traction–separation law at the interface, remains rare and appears mainly in the fibre-composite studies. Across the reviewed examples the constitutive representation is one determinant of the agreement with the bending test, but it is not the only one, since the deposited geometry and cross-section, the mesh convergence, and the boundary and contact conditions also require control. Which of these matters most depends on the response being predicted, in that the anisotropic constitutive law governs the directional stiffness, the interface law governs delamination and strength, and the modelled cross-section governs the local stress and the section stiffness.

**Figure 7:**
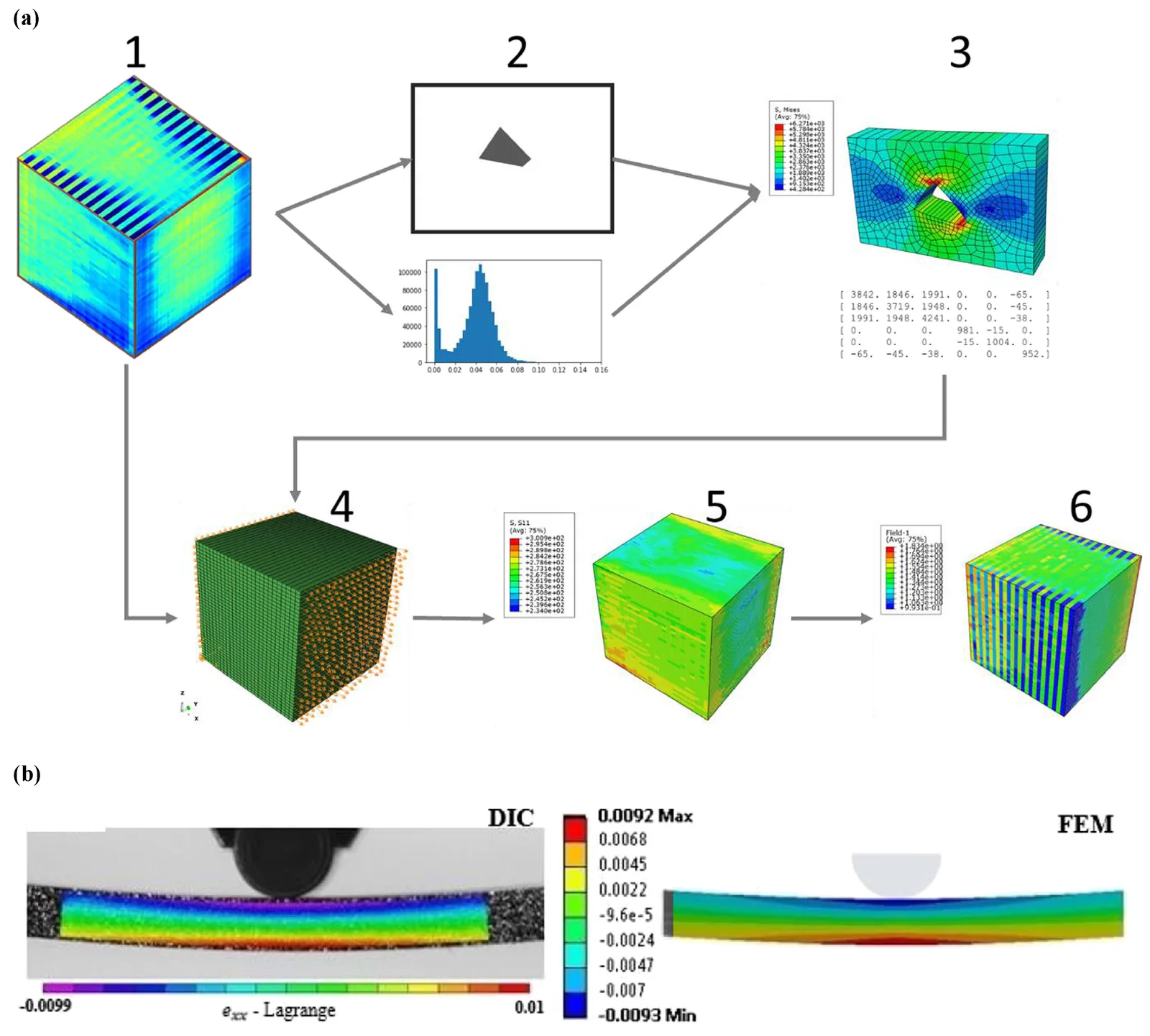
Workflow from computed tomography data to a global finite-element model (**a**) and longitudinal strain in a bending specimen measured by digital image correlation and computed with a homogenised model (**b**). The numbers 1 to 6 in (**a**) mark the steps of the method, namely processing of the computed tomography data, generation of characteristic void shapes, unit-cell responses, mapping of the unit-cell data onto the global structure, simulation of stresses and strains, and calculation of the global response and strain energy density. Reproduced from [95], Springer Nature, 2019, and adapted from [58], Springer Nature, 2022.

The coarsest of these scales, and the most economical, treats the raster as an equivalent continuum and assigns effective properties measured from tensile coupons. Driven by isotropic PLA data (E=3500 MPa, ν=0.35) obtained from tension, such models recover the elastic response across build orientations [58]. In three-point bending the strain field computed with the homogenised model agrees closely with the one measured by digital image correlation (Figure 7b) [58], while for continuous-fibre coupons a fully homogenised solid gives a first estimate of the stiffness only where the reinforcement is evenly distributed through the thickness, and the layered models that keep the reinforced layers in place come closest to the measured linear response [59]. Such models are inexpensive, yet they cannot see the individual bond between filaments, so they tend to miss the mechanisms that govern failure.

Whatever the scale, the reviewed models describe the polymer with constants fixed at room temperature and at one rate of loading. None of the 14 model setups compiled in Table 4 assigns the polymer a viscoelastic law or makes its constants depend on temperature, moisture or loading rate (counted here from the table), and one of these studies names viscoelasticity among the material models that other filaments would require in future work [157]. None of the 26 models validated against a flexural experiment does so either, as Table 5 summarises. Bending experiments show why this matters. Printed PLA tested at 50 °C instead of 25 °C lost 46% of its flexural strength, from 90.59 to 49.32 MPa, and 19% of its flexural modulus, from 2.89 to 2.35 GPa (percentages computed here) [159], and immersion in water for 720 h lowered the flexural strength of PLA by about 25% and that of PETG by about 11% [160]. Ageing acts more slowly and not always in one direction. Eight days at the onset of the glass transition lowered the flexural modulus of PLA and PETG by about 10% [160], and five days of combined heat, humidity and ultraviolet light lowered the flexural modulus of PETG from 2.35 to 1.13 GPa [161]. In contrast, ABS aged for two months in a natural environment kept its flexural strength [162], and moisture with a year of ageing weakened thermoformed PLA orthoses but not PETG ones [80]. Sterilisation in moist heat reduced the flexural modulus of printed PLA by about 30% while its flexural strength held in rectilinear infill [163]. Time under load is the clearest omission, since a PETG grade whose short-term bending stress did not change after steam sterilisation showed, after sterilisation at 121 °C, about four times the deflection of unsterilised beams after four months under constant bending load, while beams sterilised at 134 °C twisted so that their deflection could not be read reliably [164]. The rate of loading, by contrast, appears to matter little within a quasi-static test, where the flexural strength of PLA stayed between 65 and 70 MPa at crosshead speeds from 6 to 10 mm/min [165]. A bending model of printed PLA and PETG parts built on elastic constants and an apparent yield stress from tensile tests matched the measured forces within 2.25 to 5.57%, but its comparison covers only the as-printed parts, not the aged ones whose response had changed [80]. A viscoelastic description of a printed beam does exist, yet it is an analytical model of the dynamic mechanical phase shift of ABS in three-point bending, not a finite-element prediction of flexural response [166]. An elastic finite-element model is therefore a model of the dry polymer at room temperature under short loading, and its agreement with a bending test does not extend to the same part after heating, wetting or sustained load.

One scale down, mesoscale models resolve the filament and void architecture explicitly or through analytical homogenisation. A hierarchical multiscale representative volume element with asymptotic homogenisation predicts the orthotropic stiffness from raster orientation and porosity [61], and computational homogenisation of representative volume elements built from a micrograph and from micro-CT data gives orthotropic elastic constants of the printed layers that are close to transversely isotropic [15]. A related route for a uniaxially printed part goes from the void shapes measured by computed tomography, through unit-cell simulations, to a global model that returns the stiffness and the local strain-energy field (Figure 7a) [95]. Applied to curved-layer arc lattices, the same idea predicts the elastic response from the strut geometry and compares it against measurement, though the validation is tensile [168]. The logic scales to cellular parts, where a homogenised unit-cell model and a full-scale model of printed lattices are compared with cantilever bending tests on the predicted flexural rigidity [7], and lattices modelled from both the CAD and the tomography geometry predict their compressive response [6]. Data-driven variants are emerging, pairing a representative volume element with a machine-learning surrogate to estimate the infill-dependent elastic constants [60]. The same homogenisation view extends to architected lattices, where a directional homogenisation relates the effective stiffness of gyroid structures to their build orientation and the true stress is computed on CAD-derived cross-sections [140].

Homogenisation averages the interlayer plane into effective properties, yet bending fails preferentially along that plane, so interface models represent it directly. Curved composite beams have been modelled in two dimensions with a cohesive traction–separation contact whose fracture energies come from a published study of interlaminar fracture toughness and whose shear strength comes from published short-beam tests, both reduced to improve the prediction [26]. A resolved mesostructure of unidirectional parts under tensile loading, with cross-section correction factors measured from micrographs, makes the model reflect the real deposited geometry rather than the nominal one [157]. A cohesive law calibrated from double cantilever beam and end-notched flexure tests has also been placed at the interface between the two materials of printed multi-material struts, with the predicted flexural modulus compared against three-point bending [151].

All of these models start from a part that is already printed, whereas the foundational process-level models simulate deposition itself. An early three-dimensional finite-element model with element activation simulated the coupled thermal and mechanical field of the process together with the residual stress and part distortion [72], and the distortion was later reproduced in Abaqus through sequential element activation [73]. These models matter here because the residual state they predict sets the starting point for any later bending analysis. Non-planar deposition is only beginning to be modelled at this level. A multi-axis, non-planar part has been represented with a locally rotated anisotropy field on reduced-integration solid elements, one of the few models that couples the curved toolpath to the material directions it produces [158]. A further step derives the anisotropic field for both planar and non-planar components directly from the G-code, so the modelled material directions follow the deposition path as printed, with the resulting simulations validated in tensile tests with stereo digital image correlation instead of a single force reading [81].

## 6. Experimental Validation and Correction of the Finite-Element Predictions

**Figure 8:**
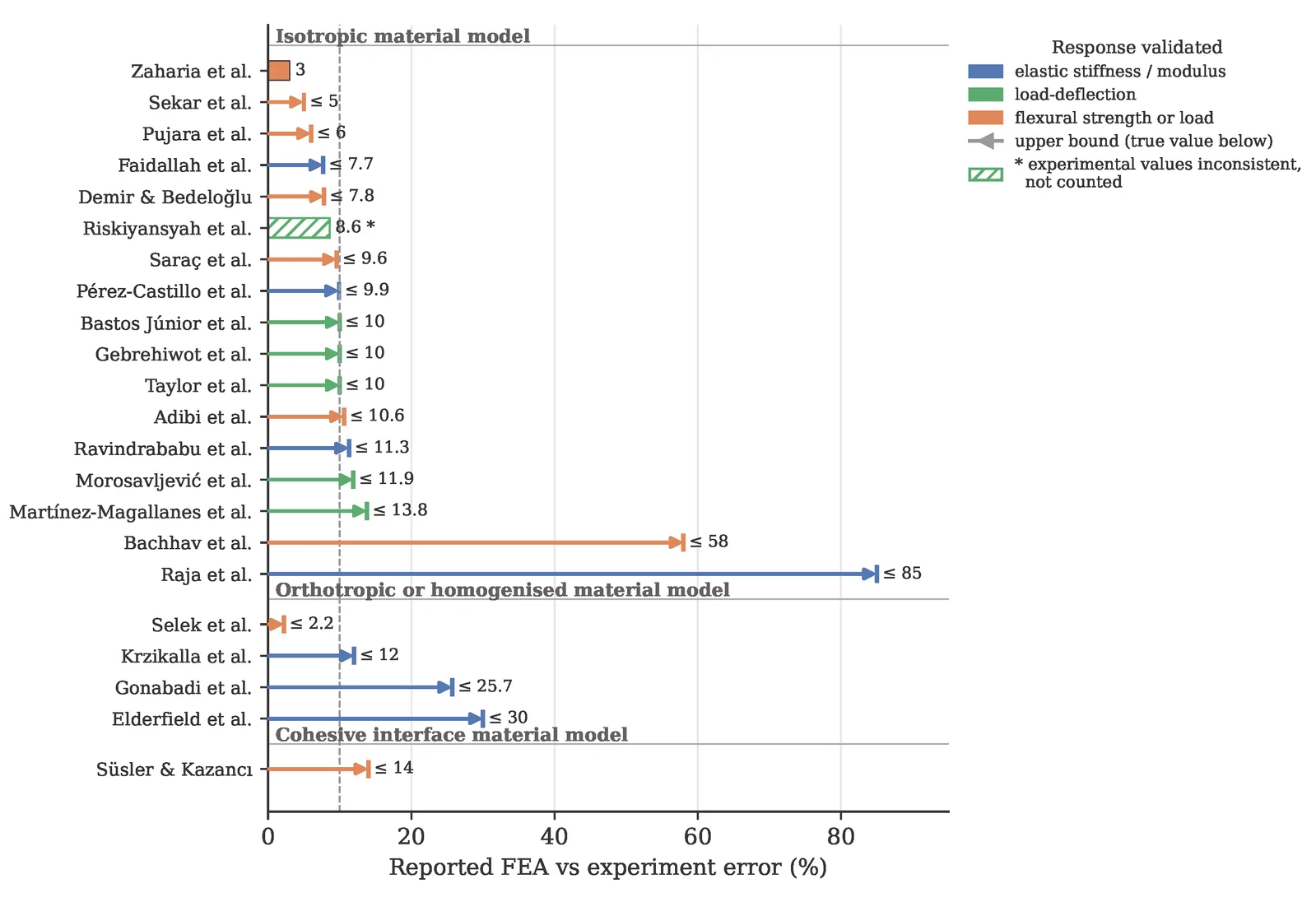
Reported finite-element versus experiment error for the flexural validations that give a numerical value, grouped by the material model of the printed material and coloured by the response quantity validated, with the study whose experimental values are internally inconsistent hatched and not counted. A filled bar marks a reported error that is not an upper bound. Studies shown [7,26,36,42,58,59,62,63,64,65,66,67,68,69,70,116,147,155,156,167,170,171].

A model is judged by how well it matches an experiment, but the match means different things for different response quantities, and agreement in the elastic range does not by itself validate a prediction of strength or of failure. Table 6 therefore groups the reviewed flexural validations by the quantity each one tests, namely the elastic stiffness, the load–deflection response, the ultimate flexural strength, and damage or delamination, and its basis column separates an independent comparison from one in which the model was calibrated against the same measurement. The errors are quoted as reported by the authors. Two of the flexural validations use a cantilever instead of three or four-point bending [7,155]. Of the 26 validations, 12 concern PLA, PETG or ABS in neat form, including the curved-layer case, and the other 14 involve fibre-reinforced or wood-filled composites, a metal-filled filament, a multi-material PLA and TPU strut, conductive TPU, Ultem 1010, or sandwich and plate-reinforced panels, so more than half of the flexural evidence for the modelling lies outside the material focus of the review. Figure 8 plots the reported errors of the 22 studies that give a numerical value, grouped by the material model and coloured by the response quantity, with the one study whose experimental values are internally inconsistent marked and not counted. Solid bars are single measured deviations, and intervals with an arrowhead are upper bounds, either a value reported as below a stated percentage or the maximum of a reported range, so the true value lies between zero and the marked limit. Validations against a non-flexural response, namely two tensile calibrations [71,157], tensile tests of curved non-planar specimens [81], a modal study [169], a creep model [153] and the compressive lattice studies [6,140], are discussed where they belong and are not pooled with the flexural errors.

### 6.1. Models That Agree

Of the 26 flexural validations, 22 report a numerical deviation and 4 report their agreement graphically. One of the 22, a comparison on midspan deflection, is marked in Figure 8 and Table 6 and left out of the counts that follow, because the experimental flexural strength stated in the same study conflicts with its own tabulated loads [170]. Of the remaining 21, 9 stay within 10% across every condition they report, and two more do so only in the elastic range or in most cases [69,167], so fewer than half of the models that give a number reproduce the measured response closely under every reported condition. The share depends on the quantity validated. Within 10% under every condition are 2 of the 7 elastic stiffness validations with a number, 1 of the 5 load–deflection validations, 6 of the 8 flexural strength or load validations and not the single damage validation. The strength group consists mostly of sandwich and plate-reinforced panels, topology-optimised specimens and a process-parameter study whose comparison concerns one maximum load or stress, while the stiffness group includes homogenised and lattice models tested across a range of architectures, so the higher rate for strength does not show that strength is the easier quantity to predict. Grouped by the material model, 8 of the 16 isotropic models and 1 of the 4 orthotropic or homogenised models with a number stay within 10%, and the one cohesive model with a number does not (Figure 8), a difference of one study at these sample sizes. For the elastic stiffness, a layered shell model and a solid model reinforced by link elements differ from the measured linear response by 3% on average and a fully homogenised one by 12% where the reinforcement is evenly distributed through the thickness [59], the flexural modulus of re-entrant honeycomb sandwich beams is predicted within 3.3 to 7.7%, computed here from the tabulated values [67], and the stiffness of both planar and curved-layer lattices with errors of 0.1 to 9.9% [42]. For solid specimens the flexural modulus is overpredicted by 6.7 to 17.1% as the build and raster orientation move away from 0° and underpredicted by 25.7% at 50% infill, values computed here from the flexural modulus of each condition printed in the source figure [58]. The agreement also depends on the architecture, with the flexural rigidity within 5 to 8% of cantilever tests at a filament spacing of about 3 mm and about 30% away at larger spacings [7]. For the load–deflection response, the deviation is 8.6% on midspan deflection [170], a comparison not counted above, within about 5% for solid coupons and within 10% for sparse ones [65], below 10% in most cases for the deflection and stress of stiffened beams whose material law comes from the same beam tests [167], below 5% from 2 to 8 mm of deflection but 7.6 and 13.8% at 1 mm for the unreinforced plates and below 10% for the reinforced ones [116], and 5.7 to 11.9% on the deflection, stress and strain of a gyroid cantilever, computed here from the tabulated values [155]. For the ultimate flexural strength, it is below 2.2% [63], about 3% [66], 1 to 6% [64], below 5% [70], 7.3 to 7.8% [147], 2.8 to 9.6% on the damage load of PLA parts strengthened with composite plates [36], 4.8 to 10.6% on the modulus and bending strength of honeycomb and auxetic cores [68], and, with calibrated interfacial values, 2 to 14% on the maximum load and 1 to 8% on the interlaminar tensile strength of curved beams [26]. Most of these are close agreements, yet they are agreements on different quantities, and none of them on its own validates a prediction of failure. Agreement in the elastic range does not guarantee agreement at failure, and one study shows this directly, with a relative error below 10% in the elastic region followed by an overprediction of the post-yield peak [69]. A model should therefore be described as validated for the quantity it was tested against, and for no more than that.

An error has meaning only against the scatter of the experiment it is compared with. Of the 26 validations, 18 state the number of specimens, between 2 and 9 per condition where it is given per condition, and only 5 report the experimental scatter numerically (Table 6). Where both are known, the comparison reads differently from study to study. For the lattices of one study the coefficient of variation of the measured stiffness is 2.9 to 36.4%, computed here from the tabulated means and standard deviations, and the model error of 0.1 to 9.9% lies below that scatter in 13 of the 16 conditions [42], and for sandwich wing sections an error of about 3% lies within a coefficient of variation of 9.8% in the measured strength [66]. In the other three the error exceeds the scatter, as an overprediction of 27 to 58% against a coefficient of variation of 1.0 to 13.0% in the measured strength [62], deviations of 4.8 to 10.6% against 1.1 to 3.1% [68], and a deviation of 12% for the fully homogenised model of evenly reinforced specimens against at most 1% in the measured modulus [59]. For the other 21 validations it cannot be said from the published data whether the reported error is distinguishable from the scatter of the test.

**Table 6:** Reported agreement between finite-element predictions and flexural experiments, grouped by the response quantity validated; specimens per condition and experimental scatter as reported, n.r. not reported in the text or tables, CV coefficient of variation, computed here where the study gives means and standard deviations.

Study	Model/Scale	Material	FEA vs. Experiment	Specimens; Scatter	Basis
*Elastic stiffness or modulus*					
Elderfield et al. [7]	Homogenised and full-scale shell	Continuous CF	5 to 8% at a filament spacing of about 3 mm; about 30% at larger spacings	n.r.	independent
Faidallah et al. [67]	Re-entrant honeycomb sandwich	Wood/PLA	3.3 to 7.7% (flexural modulus), computed here from the tabulated values	3; n.r.	independent
Gonabadi et al. [58]	Microstructural homogenisation	PLA	+6.7 to +17.1% at full infill across raster and build orientations; −25.7% at 50% infill (flexural modulus), computed here from the figure legends	5; n.r.	independent
Krzikalla et al. [59]	Homogenised solid and layered shell	CF composite	3% (layered shell and truss-reinforced solid); 12% fully homogenised, evenly distributed reinforcement	5; CV up to 1% (modulus), 7% (strength)	independent
Raja et al. [156]	Structural profiles	PLA	28 to 85% on normalised stiffness ratios; model ratios below the experimental ones for all four designs	n.r.	independent
Yavas et al. [151]	Multi-material struts, cohesive interface	PLA/TPU	agreement shown graphically; cohesive law calibrated from DCB and ENF tests	21 over 7 ratios; n.r.	calibrated
Pérez-Castillo et al. [42]	Lattice, planar and curved layers	PLA	0.1 to 9.9%	5; CV 2.9 to 36.4%	independent
Ravindrababu et al. [171]	Solid C3D8R, flat and curved specimens, planar layers	ABS	3.8 to 11.3%	3; n.r.	independent
*Load–deflection response*					
Alarifi [146]	Homogeneous isotropic unit structures	PETG/CF	agreement shown graphically, no single figure	3; n.r.	independent
Bastos Júnior et al. [69]	Topology-optimised beam	PLA	below 10% elastic; overpredicts the post-yield peak	4; n.r.	independent
Gebrehiwot et al. [167]	Beam with stiffener	PLA	below 10% in most cases (deflection, stress)	3; n.r.	calibrated
Imanian et al. [9]	Hyperelastic TPMS bending sensors	Conductive TPU	agreement shown graphically, no single figure	n.r.	independent
Martínez-Magallanes et al. [116]	Cellular and fractal plates	PLA	unreinforced plates below 5% from 2 to 8 mm of deflection, 7.6 and 13.8% at 1 mm; reinforced plates below 10%	3; n.r.	independent
Morosavljević et al. [155]	Gyroid cantilever	PLA	5.7 to 11.9% (deflection, stress, strain), computed here from the tabulated values	n.r.	independent
Riskiyansyah et al. [170]	Honeycomb rib	PLA	8.6% (midspan deflection); experimental strength values internally inconsistent, not counted	9; n.r.	independent
Scapin & Peroni [14]	Shell elements, triangular infill	Fibre-filled nylon	load against curvature compared graphically, no single figure	2; n.r.	independent
Taylor et al. [65]	Nonlinear thermo-mechanical	Ultem 1010	about 5% (solid); within 10% and 5% (sparse, two air gaps)	5; n.r.	independent
*Ultimate load or flexural strength*					
Adibi et al. [68]	Honeycomb and auxetic cores	PLA+	4.8 to 10.6% (modulus and bending strength)	3; CV 1.1 to 3.1%	independent
Bachhav et al. [62]	Resolved infill, solid tensile properties	ABS	+27 to +58% of the measured stress (overprediction), computed here; the study states 21 to 27%	5; CV 1.0 to 13.0%	independent
Demir & Bedeloğlu [147]	Auxetic-core sandwich panel	PLA core, CF/epoxy faces	7.3 to 7.8%	5; n.r.	independent
Pujara et al. [64]	Process-parameter study	Metal-filled filament	1 to 6%	n.r.	independent
Saraç et al. [36]	PLA with CFRP plates	PLA/CFRP	2.8 to 9.6% (damage load)	at least 3; n.r.	independent
Sekar et al. [70]	Topology-optimised specimens	PLA/PETG/ABS	below 5%	n.r.	independent
Selek et al. [63]	Honeycomb-core sandwich panel	ABS/CaCO_3_ core, CF/epoxy faces	0 to 2.2% (maximum bending stress)	n.r.	independent
Zaharia et al. [66]	Sandwich wing sections	Short-CF sandwich	about 3%	5; CV 9.8% (strength)	independent
*Damage, delamination or fracture*					
Süsler & Kazancı [26]	Cohesive laminate	Fibre-reinforced nylon	2 to 14% (maximum load); 1 to 8% (interlaminar tensile strength)	n.r.	calibrated

### 6.2. Models That Depart from Experiment

The largest deviations in the set have two different sources. A model that assigns one set of properties from solid tensile specimens does not represent the weaker bond between printed filaments. In one such model the infill pattern is resolved in the geometry but that bond is not, and the predicted flexural stress exceeds the measurement in all four infill and pattern combinations, by 27 to 58% of the measured value as computed here, while the study states differences of 21 to 27% [62]. The widest range, 28 to 85%, is reported on the normalised stiffness ratios of structural profiles, and there the ratios from the model lie below the experimental ones for all four designs, so a large error is not always an overprediction [156]. Outside bending, an explicit damage model of an impact test slightly overestimates the time to fracture [172]. The 4 validations without a number cannot be ranked against these, since they compare curves graphically [9,14,146,151]. In one of these overlays the printed PETG/carbon structures are modelled as homogeneous and isotropic with a single modulus and Poisson’s ratio. The model curves in the flexural stress–strain plots nevertheless pass through a peak and soften as the measured ones do, but the material law behind that response is not described in the study [146].

### 6.3. The Geometric Correction

The two reviewed studies that close a large gap do so by correcting the modelled geometry rather than by scaling the result, and both work in tension and not in bending. Calibrating a virtual raster cross-section through a Box–Behnken design lowers the finite-element error from a range of 7 to 92% down to 0.03 to 7%, but the measurements are tensile, following ASTM D638, so the result establishes the principle of geometric calibration for a material-extrusion part without itself being a flexural validation [71]. Cross-section factors measured from micrographs and then calibrated likewise bring a mesoscale model of tensile specimens into agreement [157]. Neither correction has yet been carried over to a flexural validation in the reviewed set, so the principle is established for material-extrusion parts but untested in bending. Such a calibration is also not an independent validation, and the two should not be read as interchangeable.

## 7. The Gap Between Planar and Non-Planar Finite-Element Modelling

**Figure 9:**
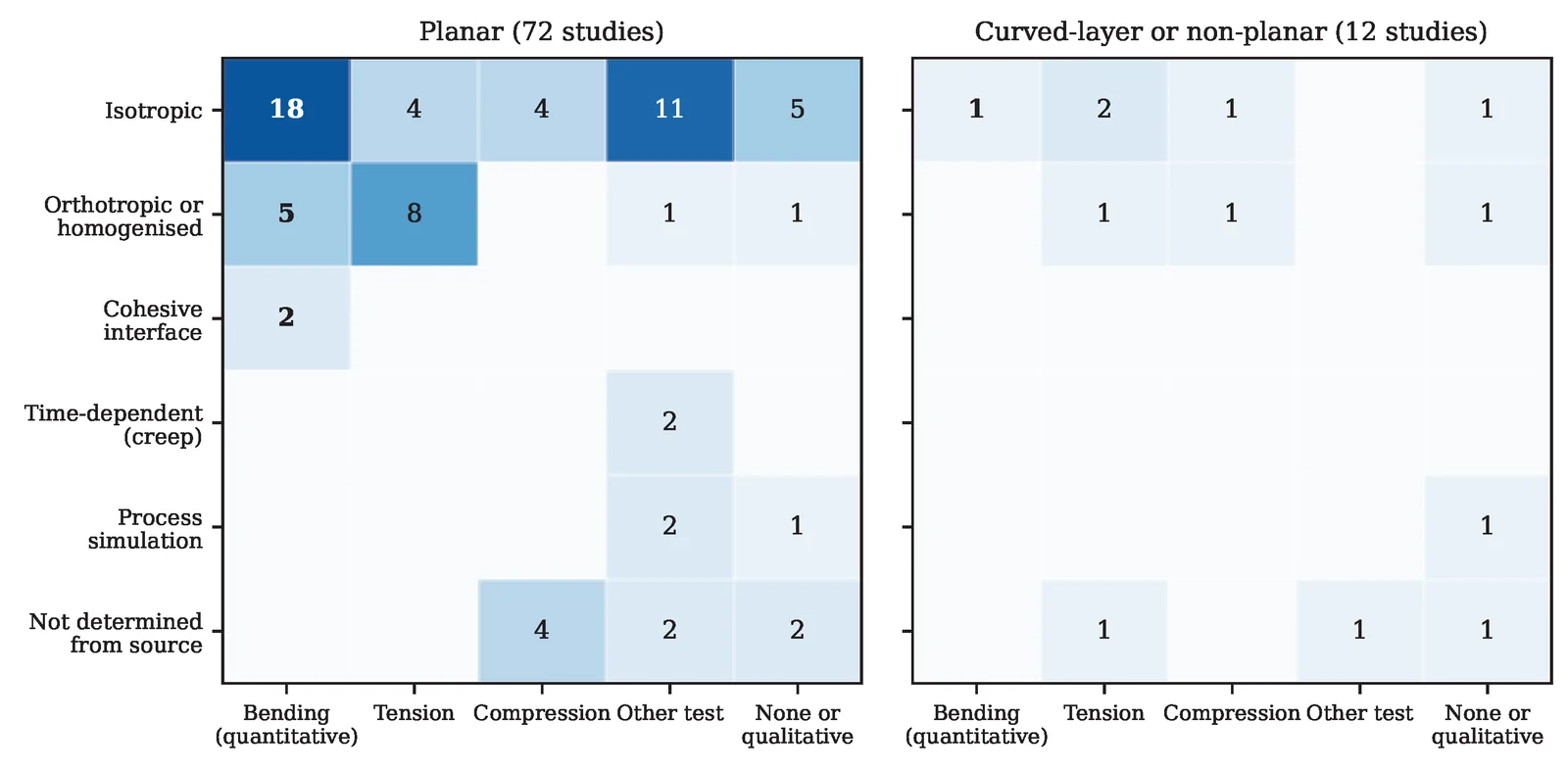
The 84 reviewed finite-element studies by deposition strategy, material model and the experiment each is compared with, in which the bending column counts the quantitative flexural validations. Each cell gives the number of studies, with darker shading for more studies, and the numbers in the bending column are set in bold.

The preceding sections leave a clear asymmetry. The largest mechanical improvements in the reviewed set come from non-planar and load-aligned deposition, as Section 4 sets out, yet 72 of the 84 finite-element studies examined in this review assume planar layers. Of the 84, 26 compare a model quantitatively with a flexural experiment, and of those 25 treat planar deposition while only 1 treats curved-layer or non-planar deposition. Of those 26, 3 are calibrated rather than independent, 2 because the interfacial properties were fitted to tests on the same material system [26,151] and 1 because its material law was taken from the same bending tests that validate it [167], and all 3 are planar, so the independent flexural evidence stands at 22 planar and 1 curved-layer or non-planar. These 26 are the studies listed in Table 6. The remaining 58 are excluded from the count because they compare the model with a non-flexural response, such as tension, compression, creep or vibration, compare it only qualitatively, or report no experimental comparison at all. The classification of all 84, with the experiment and the reason for each exclusion, is given in Supplementary Table S1, and Figure 9 shows the resulting split by deposition strategy, material model and the experiment each study is compared with. In the planar group most models assign one set of isotropic properties, and its anisotropic and homogenised models are compared mainly with tension and bending, while for 3 of the 12 curved-layer or non-planar studies the material model cannot be determined from the source.

The single validated case is a direct comparison of planar and curved-layer lattices under three-point bending [42]. A solid-element model of flat and curved ABS specimens matches the measured bending stiffness to within 3.8 to 11.3% [171], but its curved specimens were printed with planar layers on a commercial printer, and the study examines the staircase error of planar slicing, so it is counted as planar. An anisotropy built for planar and non-planar parts from the G-code has been validated against full-field measurements, but in tensile tests of curved specimens, where bending arises only because the specimen does not lie on the machine axis, so that study does not enter the flexural count [81]. A further study models exactly the comparison of interest, a planar and a non-planar tube under three-point bending with locally rotated orthotropic axes, but reports no experimental comparison [158]. The model needed for that comparison therefore exists, and the bending experiment to test it has not been reported. The larger body of planar flexural modelling, spanning homogenised coupons [58,59], lattice and cellular structures [7,116,155], cohesive interface models [26,151] and sandwich and structural analyses [66,67,68,156], has no non-planar counterpart of comparable depth.

Read against the size of each literature, the shortfall is smaller than the raw counts suggest. Non-planar and curved-layer work accounts for 12 of the 84 studies examined, and 1 of those 12 compares the model with a flexural experiment, against 25 of the 72 planar studies, a rate of 8% against 35%. Of the 12, however, 7 use the finite-element model only to compute the toolpath, and among the 5 that use it to predict a mechanical response, 1 is compared with a flexural experiment, against 25 of the 54 planar studies that do so, a rate of 20% against 46%, which at this sample size is a difference of little more than one study. Both pairs of rates also compare two samples collected with different effort, because the further searches were directed at non-planar work, so the planar denominators are lower bounds and the planar rates are uncertain in either direction. The evidence therefore does not show that non-planar modelling is held to a lower standard. The shortage lies in the number of non-planar modelling studies that predict a mechanical response at all. The aggregate review of the field is consistent with this, as finite-element analysis enters the non-planar work mainly as the means of computing the toolpath, and the cases computed that way average an 80% improvement, against 49.2% across all the studies [79], while the prediction of the resulting response is rarely the quantity being checked. The record-by-record check of the Scopus and Web of Science returns reported in Section 2.2 found no further study that pairs a finite-element model of non-planar deposition with a bending experiment, which indicates that this scarcity is a property of the field rather than an artefact of the search. In absolute terms, a single validated case is too few to support a prediction of the flexural behaviour of the deposition strategy that delivers the largest reported improvements, and it is a curved-layer lattice printed on a three-axis machine, so no multi-axis non-planar part has yet been validated in bending.

Two obstacles explain the asymmetry, one in the representation and one in the experiment. A non-planar toolpath produces a material whose principal directions rotate through the part, and few solvers accept that field as an input. It is exactly this rotation that has been represented explicitly through locally rotated material axes [158] and reconstructed from the deposition path itself [81]. The experimental obstacle is the more easily overlooked. A non-planar part is rarely a flat coupon, so the specimen geometry departs from the standard bending configuration, and when curved specimens are loaded in tension, the geometry adds bending and the force reading of a testing machine can itself be compromised, which is why stereo digital image correlation has been used as the more reliable basis for validation [81]. The scarcity of validated non-planar flexural models is therefore not only a matter of modelling effort, since the standard measurement on which planar validation rests does not transfer directly to a curved specimen.

Closing the gap means generalising the anisotropic description to the curved toolpaths that improve the mechanical response most, and pairing it with a measurement suited to a non-flat specimen. The study that would settle it is one in which a single flexural geometry is printed and modelled in both planar and non-planar form, with the two predictions validated against the same experiment, so that the deposition strategy is the only variable that differs.

## 8. Limitations of the Method

The counts reported here describe the reviewed set and not the whole field. The corpus was assembled from the returns of an open bibliographic index rather than a subscription abstracting service, so a poorly indexed study may be under-represented. The five queries were therefore re-run on both Scopus and Web of Science, as Table 1 reports, and every record they returned on the query that carries the central claim is either cited here or lies outside the scope. Requiring a digital object identifier made every record verifiable against Crossref, but it is a bibliographic and not a methodological criterion, and a relevant study published without one would have been missed. The further searches were directed at non-planar deposition, so the planar finite-element set is a lower bound.

The screening was limited as well, in that the title and abstract stage was worked through from a relevance ranking, so it was not an exhaustive reading of every record, and the full-text stage covered the candidates whose full text was obtained. The classification of the finite-element studies that carries the central claim was made from the full text of each study, and every decision is recorded with the experiment behind it and a verbatim quotation from the study. It was checked in full by two further authors, but not independently, so only the raw agreement is given. No formal appraisal of study quality or risk of bias was made. The quality attributes recorded are whether a finite-element comparison is independent of the data used to calibrate the model (Table 6), the number of specimens and the experimental scatter of each validation and, for the studies behind the extracted values, whether they state how many specimens were tested, and the synthesis does not weight the studies by them.

For the 26 flexural validations that carry the central count, a short appraisal of the sources follows from what they report. Three appear in conference proceedings [9,62,69] and the others in journals, 18 state the number of specimens, 5 report the experimental scatter numerically, and one is internally inconsistent in its experimental values [170]. Removing the three proceedings papers and the inconsistent study leaves 22 validations, 21 planar and the same curved-layer one, and 9 of the 19 among them that give a usable number stay within 10%, against 9 of 21 in the full set, so neither the deposition split nor the agreement rate rests on these sources. Removing in addition the three validations published in a university journal and in two independently published journals [63,64,147] leaves 19 validations, 18 planar and 1 curved-layer, with 6 of the 16 that give a usable number within 10%, so the agreement rate falls slightly and the deposition split is unchanged.

Comparison across the primary studies is limited by how they report. They use different standards, geometries and conditions, which limits the direct comparison of absolute values and is why the quantitative displays are read here for their trends and their spread rather than as a ranking. The extraction also exposed how unevenly flexural results are reported. Of the 21 studies behind the extracted values, 6 do not state how many specimens were tested, no value was digitised from a plotted curve, a value a study shows only in a plot was recorded as not reported, and the values of one study were read from a table printed as an image. In one study, the text reports 53.89 MPa while its own summary table gives 10.35 MPa, the value that its tabulated loads of about 275 N over a 150 mm span on a section 60 mm wide and 10 mm deep give under the ASTM D790 expression the study adopts [170]. Its measured values were therefore left out of the extracted set rather than reconciled by choosing one of the two numbers. The model-to-experiment comparison from that study, which concerns the midspan deflection and not the strength, is kept in Table 6 but marked and left out of the agreement counts, because the reliability of the other experimental data of the study is in doubt.

Whether the reported spread depends on weakly indexed venues was tested directly, since several of the highest PLA and PLA+ values come from such sources. Removing all of them leaves 43 of the 60 PLA and PLA+ conditions, lowers the maximum from 103.1 to 90.0 MPa, and leaves the other two polymers unchanged. The range for PLA and PLA+ is then 10.0 to 90.0 MPa, still close to a factor of nine, so the wide spread on which the synthesis rests is a property of the field and not of a few weakly indexed reports, and those values are therefore retained. The within-study ratios of Figure 1 and Figure 4 do not rest on them either, since without the two weakly indexed sources among them all 11 upright-to-flat ratios of the one study that varies the build orientation lie below 1 and 8 of the 10 infill series remain [13,62].

The central count also depends on where the boundary is drawn, and the boundary used here is deliberately strict, in that a study enters the flexural corpus only when it pairs a finite-element model with an experiment that loads the part in bending. Under that criterion the split is 25 planar to 1 non-planar. Tightening it to require a standard three or four-point configuration removes the two cantilever tests, both planar, leaving 23 planar to 1 non-planar. The non-planar share is therefore 4% under both the criterion used and the stricter one, and loosening the criterion instead to admit a tensile test in which a curved specimen also bends would add one non-planar study [81] and raise the share only to 7%, so the asymmetry does not depend on this choice. That record gives the experiment behind every classification, so a reader who prefers a different boundary can recount the corpus from Supplementary Table S1.

The search also bounds what can be said about phase-field fracture. Phase-field fracture was included among the numerical modelling terms of query S4, and the search returned 17 records carrying it in the title, of which 7 concern additive manufacturing. Of those 7, 2 model thermal or morphological evolution during deposition and not the mechanical response, 2 treat metallic systems, and 3 do model fracture in printed polymers. None of the 3, however, compares the model against a flexural experiment, which is what the eligibility rule of Section 2.2 requires, so no phase-field study enters the set of validated flexural models. Fracture in printed polymers is at present modelled almost entirely with cohesive interfaces and element deletion, where the crack path is confined to planes chosen in advance, and not with formulations that let the crack find its own path. Because the interlayer plane is precisely where a printed beam fails, and its position is known while its competition with through-filament fracture is not, this is a methodological gap alongside the one in non-planar deposition.

## 9. Conclusions

The flexural response of a material-extrusion part is governed by its mesostructure, and the three research lines reviewed here act on that mesostructure in sequence. The process parameters set the anisotropy and the interlayer bond, the toolpath orients the filaments with respect to the load and fixes the surface geometry, and the finite-element model must represent both to predict the bending behaviour. Within the mesostructure the build orientation has the larger effect, since an upright build is the weakest at every infill density and at high density lowers the flexural strength by a factor of more than two, whereas the raster angle changes it by 6.9% once the section is solid [12,13], and infill density has a strong but not dominant influence. The toolpath, in turn, is a primary mechanical variable. Changing the deposition direction alters the flexural response by amounts comparable to the largest reported process-parameter effects [41,42,82], but the direction of that change depends on the geometry and the loading, as the stiffer planar domes and the null result for the strong orientation both show [121,131]. A ranking of toolpath against process parameters is not available from a corpus whose conditions are not comparable, and none is claimed here. On the modelling side, 9 of the 21 flexural validations that report a usable number agree within 10% under every reported condition. The share depends on the response quantity validated, and of the five validations that report the experimental scatter, two have errors within it and three beyond it. The largest deviations come from models with properties of solid tensile specimens and from the stiffness ratios of structural profiles [62,156], and the corrections that close a large gap by adjusting the modelled cross-section have so far been demonstrated in tension only [71,157]. The least developed step is the modelling of the toolpath. Within the identified literature the deposition strategy with the largest reported effects, non-planar printing, is the one with the least numerical support [42,81,158], with 1 curved-layer validation in bending against 25 planar. The shortfall lies in the small number of non-planar studies that predict a mechanical response, not in a lower standard of validation. The validated models also share their assumptions, since none makes the polymer constants depend on temperature, moisture or loading rate, none gives the bond between layers of one polymer its own interface law, and 20 of the 26 assign one set of isotropic properties to the printed material. The thermal origin of the bond, which forms by neck growth and molecular diffusion while the deposited polymer stays hot [96,97], explains why heat treatment acts on the flexural response in a direction that depends on the polymer and the treatment temperature. Annealing raised the flexural strength of PLA at 60 °C [105], left that of ABS within a narrow band between 100 and 135 °C [106] and lowered the flexural modulus of PETG at 190 °C [107].

For modelling, a model that predicts the elastic response of a printed beam well should not be presented as validated for its strength, because the reviewed studies show these two agreements coming apart, and a scalar factor fitted to close the remaining difference improves the agreement without explaining its cause. For the experiment, a non-planar specimen is not a standard coupon, and the reviewed work indicates that a single force reading may not be a sufficient basis for validating it [81], so full-field measurement should be part of the test plan of such a study from the outset.

Modelling and validating the flexural response of non-planar parts with the rigour now applied to planar ones is therefore the clearest direction for future work. What is required is a study in which one flexural geometry is printed in both planar and non-planar form, modelled with an anisotropic description tied to the deposition path, and compared against a measurement suited to a specimen that is not flat.

## Figures and Tables

**Table 1:** Records returned by the five queries in Scopus, Web of Science and OpenAlex.

Query	Concepts Combined	Scopus	WoS	OpenAlex
S1	non-planar deposition × process	159	136	176
S2	flexural response × process × parameters	760	654	477
S3	interlayer bonding × process	1027	1018	910
S4	numerical modelling × process	1972	1896	1373
S5	S1 × mechanical response	36	35	47

**Table 2:** Representative measured flexural properties from the reviewed corpus.

Study	Material	Key Conditions	$\sigma_{f}$ (MPa)	$E_{f}$ (GPa)	Standard
*Neat polymers (focus set)*					
Boztepe [12]	PLA+	100% infill, 0° raster	101.82	3.10	ASTM D790 [117]
Sekar et al. [70]	PLA/PETG/ABS	full density, 0/90 raster	90/85/80	n.r.	ASTM D790
Saravana Kumar et al. [25]	PLA	Taguchi optimum	78.52	n.r.	ASTM D790
Hussein & Aboud [111]	PETG	three-point	70.62	1.72	ASTM D790
Alkabbanie et al. [98]	PLA	190 °C extrusion	59.06	n.r.	ASTM D790
Buss et al. [13]	PLA	90% cubic, flat build	30.73	n.r.	ISO 178 [118]
Bachhav et al. [62]	ABS	70% triangular	30.58	n.r.	ASTM D790
*Reinforced or blended materials*					
Krzikalla et al. [59]	CF composite	specimen E-0.29, reinforced layers evenly distributed	147.6	12.59	ASTM D790
Ibrahim & Hasan [33]	PLA-CF	gyroid 70%, optimum	72.6	n.r.	ASTM D790
Deivasikamani et al. [35]	PETG + CF	2.4 mm shell	52.96	2.46	ASTM D790
Zheng et al. [38]	PLA biocomposite	15% wood filler	69.07	2.57	ISO 178

n.r., not reported.

**Table 3:** Reported changes in mechanical response from non-planar, load-aligned and stress-aligned deposition, grouped by mechanical test.

Study	Approach	Reinforcement	Reported Change vs. Baseline
*Flexural/bending*			
Ding et al. [41]	Non-planar slicing, 3-axis	Continuous CF	+170% maximum bending force, −63% roughness (vs. planar)
Pierre et al. [82]	Non-planar curved beams, 6-DOF robot	CF PEEK	+28% effective bending stiffness, −31% surface deviation (vs. planar curved beam)
*In-plane fill grading (not a deposition strategy)*			
Liu et al. [54]	Adaptive-density infill, planar layers	Neat	+140% maximum flexural load, +47% ultimate compression load, +82% maximum impact resistance (vs. uniform infill)
*Impact*			
Palmer & Laliberté [40]	Non-planar slicing	PETG, neat and CF	+65% average impact (vs. planar)
Franco & Czekanski [121]	Non-planar sinusoidal	Neat	+50.6% for the weak orientation, no significant change for the strong one
*Tensile*			
Beaumont et al. [126]	Multi-axis extrusion	Continuous CF	190.76 vs. 60.31 MPa fibre-direction tensile; a comparison between two material systems, not a flexural improvement from the toolpath alone
Sebbe et al. [84]	Non-planar layers, modified 3-axis printer	Neat	at 10°, +11% (flat) and +24% (upright) tensile strength; at 30°, −21% and +5% (vs planar), computed here from the tabulated strengths
Allum et al. [130]	Non-planar zigzag toolpaths	Neat	up to +62% strength and +245% toughness in the Z direction, single-filament tensile specimens (vs. optimised planar)
Fang et al. [56]	Curved slicing, stress-aligned	Continuous CF	failure load up to +644%, stiffness up to +240%, both in tension (vs. planar layers)
Kubalak et al. [129]	Multi-axis surface reinforcement	Neat	+59% yield strength and +9% tensile modulus with a single reinforcing skin (vs unskinned bars)
*Shear*			
Xu et al. [128]	Interlaced bio-inspired paths	Neat	+31.9% and +67.5% shear strength for two schemes (vs. planar toolpath)
*Compression/modelled stress*			
Nayyeri et al. [127]	Non-planar stress-aligned (META)	Neat	up to −31% von Mises stress (vs. planar)
Castro-Avilés et al. [131]	Curved-layer thin shells (domes)	Neat	planar stiffer than curved by 16% (4 mm) and 24% (2 mm)
*Structural component test*			
Guidetti et al. [83]	Stress-flow-aligned, 5-axis	Liquid-crystal polymer	failure strength ×44 and stiffness ×6 (vs. conventional printing); a bracket, not a standard coupon
Sales et al. [53]	Principal-stress-line toolpath	Neat	+50% maximum load in an in-plane cantilever test over uniform infill patterns of the same mass

**Table 4:** Finite-element model setup across representative studies, ordered by how the interlayer anisotropy is represented.

Study	Element Type	Material Model/Inputs	Interlayer/Delamination	Software
*Explicit interface or failure model*				
Süsler & Kazancı [26]	2D surface-based cohesive	In-plane elastic constants of UD carbon/nylon and Onyx laminae	Traction–separation cohesive contact between layers	Abaqus/CAE 2021
Verma et al. [21]	CPS4R plane stress	Transversely isotropic elastic	Hashin failure criterion	Abaqus
Seibel & Kiendl [157]	8-node solid	Linear isotropic elastic, brittle failure, per strand	Element birth-and-death; cross-section factors	Ansys Mechanical 22.2
*Orthotropic or homogenised (anisotropy via effective properties)*				
Sheikh & Behdinan [61]	Linear tetrahedra (meshed in Ansys)	Orthotropic homogenised (RVE)	Periodic boundaries; no explicit interface	Ansys, MATLAB
Somireddy & Czekanski [15]	Linear tetrahedra (RVE)	Orthotropic layers, found transversely isotropic	Implicit in constitutive law	HyperMesh (mesh); solver n.r.
Bonada et al. [60]	SOLID187 (10-node)	Orthotropic homogenised (RVE + ML)	Periodic RVE geometry; no explicit interface	Ansys
Selek et al. [63]	n.r. (refined mesh)	Orthotropic ($E_{1}\;=\;E_{2}$, $E_{3}$) from tension	Implicit in constitutive law	ANSYS
Ramful [154]	Hexahedra	Thermal process model of warping	First layer to build plate interface	FEMAP, LS-DYNA
*Non-planar anisotropy*				
Piatkovsky & Tashkinov [158]	C3D8R (8-node)	Orthotropic with locally rotated axes	Anisotropy follows the curved wall	Abaqus
*Calibrated deposited geometry (planar, tensile calibration)*				
Hachimi et al. [71]	C3D4 tetrahedra	Monofilament tensile properties, calibrated raster section	Virtual raster cross-section, calibrated against ASTM D638 tension	Abaqus
*One set of material properties (anisotropy neglected)*				
Bachhav et al. [62]	Tetrahedra, 1 mm	Nonlinear, from solid tensile specimens (D638); infill resolved	None	Ansys
Raja et al. [156]	Tetrahedra (default mesh)	Isotropic PLA (60 MPa, 3.5 GPa)	None	ANSYS Workbench 2024 R2
Bastos Júnior et al. [69]	Hexahedra (solid), TET10 (optimised)	Multilinear isotropic plasticity, PLA	None	Ansys 2025 R1
Morosavljević et al. [155]	Quadratic tetrahedra	PLA, resolved gyroid geometry	None (cellular architecture)	nTopology

**Table 5:** Modelling capabilities of the 26 finite-element models compared quantitatively with a flexural experiment, counted here from the model descriptions of the studies.

Capability	Models	Studies
Anisotropy of the printed material in the constitutive law (orthotropic, transversely isotropic or homogenised)	6	[7,14,26,58,59,63]
One set of isotropic properties for the printed material	20	the remaining models; the cellular or sparse architecture is resolved in the geometry where present
Different properties in tension and compression	1	[65]
Interface law between layers of one polymer	0	none
Cohesive interface law between two materials	2	[26,151]
Nonlinear material response (plasticity or hyperelasticity)	9	[9,26,62,65,66,68,69,151,167]
Failure or damage criterion	3	[26,36,151]
Temperature-dependent constants	0	none; one study tests at six temperatures but simulates room temperature only [65]
Moisture-dependent constants	0	none
Rate dependence or viscoelasticity	0	none

## Data Availability

The classification of the finite-element studies is provided in Supplementary Table S1. Other data presented in this study are available on request from the corresponding author.

## Supplementary Materials

The following supporting information can be downloaded at https://www.mdpi.com/article/10.3390/polym18202604/s1, Table S1: Classification of the 84 cited primary studies that build their own finite-element model of a printed part. References [1,2,3,4,5,6,7,9,10,11,14,15,19,21,22,26,30,31,36,38,42,46,50,53,55,56,58,59,60,61,62,63,64,65,66,67,68,69,70,71,72,73,81,83,85,89,94,95,99,101,111,115,116,127,131,132,137,138,139,140,141,142,143,144,145,146,147,148,149,150,151,152,153,154,155,156,157,158,167,168,169,170,171,172] are cited in the Supplementary Materials.

## Abbreviations

The following abbreviations are used in this manuscript:

ABS	Acrylonitrile butadiene styrene
CF	Carbon fibre
FDM	Fused deposition modelling
FEA	Finite element analysis
FFF	Fused filament fabrication
PETG	Polyethylene terephthalate glycol
PLA	Polylactic acid
RVE	Representative volume element
